# Wheat ocs-Element Binding Factor 1 Enhances Thermotolerance by Modulating the Heat Stress Response Pathway

**DOI:** 10.3389/fpls.2022.914363

**Published:** 2022-05-31

**Authors:** Harsha Samtani, Aishwarye Sharma, Paramjit Khurana

**Affiliations:** Department of Plant Molecular Biology, University of Delhi South Campus, New Delhi, India

**Keywords:** bZIP, endoplasmic reticulum (ER), heat stress, nucleus, ocs-element binding factor 1 (OBF1), thermotolerance, wheat

## Abstract

The basic leucine zipper family (bZIP) represents one of the largest families of transcription factors that play an important role in plant responses to abiotic stresses. However, their role in contributing to thermotolerance in plants is not well explored. In this article, two homoeologs of wheat ocs-element binding factor 1 (TaOBF1-5B and TaOBF1-5D) were found to be heat-responsive TabZIP members. Their expression analysis in Indian wheat cultivars revealed their differential expression pattern and TaOBF1-5B was found to be more receptive to heat stress. Consistent with this, the heterologous overexpression of TaOBF1-5B in *Arabidopsis thaliana* and *Oryza sativa* promoted the expression of stress-responsive genes, which contributed to thermotolerance in transgenic plants. TaOBF1-5B was seen to interact with TaHSP90 in the nucleus and TaSTI in the nucleolus and the ER. Thus, the results suggest that TaOBF1-5B might play an important regulatory role in the heat stress response and is a major factor governing thermotolerance in plants.

## Introduction

Transcription factors function as important components of regulatory networks present inside the cell. They modulate the expression of the target genes by binding to specific sequences in their promoter region (Kong et al., 2015; Yamaguchi, 2021). Amongst the various transcription factor families in plants, basic leucine zipper family (bZIP) represents one of the family, which participates in regulating diverse biological functions, such as stress and hormone signaling, floral induction and development, seed maturation and germination, photomorphogenesis, and pathogen defense (Jakoby et al., 2002; Nijhawan et al., 2008).

bZIP proteins possess the characteristic bZIP domain composed of the basic region and a leucine zipper (Dröge-Laser and Weiste, 2018). The bZIP domain spans over 60–80 amino acids. The basic region consists of 16 amino acids which are further characterized by the presence of an invariant N-x_7_-R/K motif. The leucine zipper is composed of a heptad repeat of leucine or other hydrophobic amino acids, such as isoleucine, valine, methionine, and phenylalanine, situated nine amino acids toward the C-terminal. While the basic region binds to DNA in a sequence-specific manner, the leucine zipper is in the form of a coiled-coil amphipathic sequence, which is less conserved and determines dimerization aspects, i.e., homodimerization and heterodimerization (Nijhawan et al., 2008; Dröge-Laser et al., 2018).

bZIP is one of the most diverse proteins and is reported in many eukaryotes. Several members of this family have been reported in organisms, such as *Arabidopsis* (75), rice (89), human (56), *Sorghum* (92), Soybean (131) and maize (125), *Brachipodium distachyon* (96), *Triticum aestivum* (191), *Musa acuminata* (121), and *Manihot esculanta* (77) (Riechmann et al., 2000; Deppmann et al., 2004; Liao et al., 2008; Nijhawan et al., 2008; Wang et al., 2011; Wei et al., 2012; Liu and Chu, 2015; Hu et al., 2016a,b; Agarwal et al., 2019). Studies suggest that plant bZIPs can bind to DNA sequences with an ACGT core *cis*-element, such as ABRE, G-box (CACGTG), C-box (GACGTC), A-box (TACGTA), T-box (AACGTT), and a GCN4 motif [TGA (G/C)TCA] (Foster et al., 1994; Agarwal et al., 2019).

Several bZIPs have been implicated to play an important role in abiotic stresses, such as drought stress, saline stress, and cold stress. In the case of *Arabidopsis, AtbZIP17* and *AtbZIP24* were reported to enhance salt tolerance by regulating the expression of stress-responsive genes (Liu et al., 2008; Yang et al., 2009). *AtbZIP1* was found to be upregulated by cold, salt, and drought stress and its overexpression promoted salt and drought tolerance, thereby making it a positive regulator of the stress response (Sun et al., 2012). *AtbZIP62* has been reported as a negative regulator of salt stress response in *Arabidopsis thaliana* (Rolly et al., 2020). In the case of rice, overexpression of *OsbZIP23* in rice plants significantly enhanced drought and salt tolerance and led to increased ABA sensitivity (Xiang et al., 2008). Similarly, Lu et al. (2009) reported the role of *OsbZIP72* in ABA signaling and drought tolerance. OsbZIP52 was found to function as a negative regulator in cold and drought stress, as the rice plants overexpressing *OsbZIP52*, were found to be cold and drought-sensitive, and various stress-regulated genes were observed to be down-regulated in the transgenic plants (Liu et al., 2012). The role of *OsbZIP39* was reported in regulating the ER stress response, as its overexpression enhanced the expression of ER stress marker genes (Takahashi et al., 2012).

As compared to *Arabidopsis* and *Oryza sativa*, there have been very limited reports elucidating the functional role of *TabZIPs*. In wheat, overexpression of *TabZIP60* enhanced multiple stress tolerance in *Arabidopsis* through the ABA signaling pathway (Zhang et al., 2015). Further, the splicing of *TabZIP60* was found to provide heat stress tolerance in *Arabidopsis* plants by regulating the expression of ER stress-related genes (Geng et al., 2018). Similarly, *TabZIP14-B* was found to participate in freezing and salt tolerance in transgenic *Arabidopsis* plants (Zhang et al., 2017). Agarwal et al. (2019) identified *TabZIP*, which provided tolerance against environmental stress, such as heat, salinity, and dehydration. *TabZIP6* was identified to negatively regulate cold stress response, as it was found to bind to CBF and decrease the expression of downstream COR genes in *Arabidopsis* overexpression lines (Cai et al., 2018).

*Ocs*-element binding factor (OBF) proteins belong to group S bZIPs, which bind to promoters containing 20 bp element named as *ocs* (Katagiri and Chua, 1992; Zhang et al., 1993). This *cis*-acting *ocs*-element was first discovered in the promoter of the octopine synthase (*ocs*) gene of *A. tumefaciens* (Zhang et al., 1993). The proteins that belong to the OBF bZIP subfamily are also classified as TGA transfactors (Rieping et al., 1994). Maize OBF1 was reported to accumulate in response to low temperature (Kusano et al., 1995). In the case of *O. sativa*, OsOBF1 has been identified as an interacting protein of low-temperature induced lip19 protein, although the transcripts of *OsOBF1* were found to decrease under low-temperature treatment. Thus, *OsOBF1* is speculated to be involved in a low-temperature signal switching mechanism in rice (Shimizu et al., 2005). Similarly, in the case of wheat, the transcript of *TaOBF1* was found to increase under cold and drought stress, and TaOBF1 heterodimerized with Wlip19 (wheat lip19 homoeolog) (Kobayashi et al., 2008). However, till now TaOBF1 has not been functionally characterized in any plant.

In this study, we have identified *TaOBF1-5B* as a heat-responsive bZIP. Its interaction with TaSTI and TaHSP90 suggested its potential role in heat stress response. Overexpression of *TaOBF1-5B* in both *Arabidopsis* and *O. sativa* provided thermotolerance by increasing the expression of stress-responsive genes. Thus, this data suggests that apart from being involved in low-temperature response, *TaOBF1* might also play an important role under heat stress conditions.

## Materials and Methods

### Phylogenetic Analysis

The protein sequences of all the OBF1 members were downloaded from the NCBI database. The multiple sequence alignment was performed by using the MUSCLE program and the neighbor-joining (NJ) phylogenetic tree was constructed by using MEGA 7 software.

### Expression Analysis of *TaOBF1* in *Triticum aestivum*

For analyzing the expression of *TaOBF1-5B* and *TaOBF1-5D* in various developmental stages, the wheat expression database hosted at http://wheatexpression.com was used (Borrill et al., 2016). As this database harbored the normalized data, therefore, transcripts per kilobase millions (TPM) values were Log transformed (Log_2_X) to generate a heatmap using gplot package and Rcolorbrewer package. For seedling-based studies, wheat plants of Indian cultivar PBW-343 and K7903 were germinated on a cotton bed in sterile petri-dishes in a growth chamber maintained at 24/20°C temperature under a daily cycle of 16 h light/8 h dark photoperiod, having 200–300 μmol m^−2^ s^−1^ of light intensity. For studying the effect of high temperature, the seedlings of K7903 and PBW-343 cultivar were kept in a growth chamber, that was set at 30, 35, 40, and 45°C and were collected at 2 hrs after the stress treatment (Agarwal and Khurana, 2019). For checking the expression of *TaOBF1* homoeologs in various tissues, plants of PBW-343 were grown in potting soil in the departmental net house. For heat stress treatment, the plants were transferred for 4 h to a growth chamber maintained at 42°C for 5, 10, 15, and 20 days after anthesis (Agarwal and Khurana, 2019). A sampling of tissues like root, flag leaf, anther, ovary, lodicule, and developing grain was done from the spike of the control and stressed plants simultaneously. The tissue was immediately frozen in liquid nitrogen and stored at −80°C until RNA isolation.

### Transactivation Assay

For checking the transactivation of TaOBF1-5B, the full-length CDS was subcloned into the GAL4 DNA-binding domain of the pGBKT7 vector (Clonetech, USA) by using the Gateway™ cloning technology (Directional TOPO cloning kit and LR clonase Enzyme mix II kit, Invitrogen Inc., USA). The recombinant plasmids were then transformed into yeast strain AH109 harboring the HIS3 reporter gene. The transformants were selected on tryptophan lacking media (SD-W) and the transactivation activity was screened by dropout assay performed on tryptophan and histidine lacking media (SD-HW).

### Subcellular Localization and BiFC Assay

To determine the subcellular localization of TaOBF1-5B, the full-length CDS was amplified from the 2 h heat stress cDNA, using the HF phusion enzyme (NEB), and then cloned into the pENTR/D-TOPO vector. It was then mobilized into the destination vector, i.e., pSITE3CA, having the CaMV35S promoter (Directional TOPO cloning kit and LR clonase Enzyme mix II kit, Invitrogen Inc., USA). The ORF of the gene was fused in frame with the C terminal of YFP. This plasmid construct was then used for coating the gold particles and bombarded at a pressure of 1,100 psi into the onion epidermal peel cells by using the PDS-1000 bombardment system (Bio-Rad, Canada). Transformed onion peels were incubated at 27°C for 16 h under dark conditions and the fluorescence was observed in the confocal microscope (Leica, Germany).

For the BiFC assay, the CDS of *TaHSP90* and *TaSTI-2A* were first cloned into pENTR/D-TOPO vector and then mobilized into pSITE-cEYFP. Similarly, the CDS of *TaOBF1-5B* was cloned into pSITE-nEYFP. The plasmid combinations were used for coating the gold particles and then bombarded at a pressure of 1,100 psi into the onion epidermal peel cells by using the PDS-1000 bombardment system (Bio-Rad, Canada). Transformed onion peels were incubated at 27°C for 16 h under dark conditions and the fluorescence was observed in the confocal microscope (Leica, Germany).

For checking the interaction between putative interactors, the genes of *TaOBF1-5B, TaOBF1-5D, TaHSP90*, and *TaSTI-2A* were cloned into the pENTR/D-TOPO vector and further into pDEST-GADT7 and pDEST-GBKT7 vectors (Clonetech, United States). The plasmid combinations were transformed into the yeast AH109 strain harboring the HIS3 reporter gene. The reporter gene activity was confirmed by the viability test on a medium lacking histidine, leucine, and tryptophan (-HLW) supplemented with 0.5 mM 3AT (3-Amino-1,2,4-triazole).

### *TaOBF1-5B* Cloning and Overexpression in *Arabidopsis thaliana*

For generating the *TaOBF1-5B* overexpression lines in *Arabidopsis*, the full-length CDS was amplified and cloned into the pENTR/D-TOPO vector. It was then mobilized into a pMDC32 destination vector having the CaMV35S promoter using the Gateway™ cloning technology (Directional TOPO cloning kit and LR clonase Enzyme mix II kit, Invitrogen Inc., USA). The EHA105 strain of *Agrobacterium tumefaciens* harboring the pMDC32-*TaOBF1-5B* was used for the transformation of *Arabidopsis* plants by the floral dip method (Zhang et al., 2006). Positive transgenic plants were selected after successive generations of selection on MS-agar medium supplemented with 50 μg/μl of hygromycin. The plants were further confirmed by PCR by using the hygromycin and gene-specific primers. Members of the T_3_ generation with 100% resistance to hygromycin were considered to be homozygous. The level of ectopic expression in homozygous plants was checked by RT-PCR analysis.

### *TaOBF1-5B* Overexpression in *Oryza sativa*

For generating the *TaOBF1-5B* overexpression lines in rice, the full length CDS was cloned into pIPKb002 gateway-based vector under the control of maize ubiquitin promoter (Himmelbach et al., 2007; accession no - EU161568). This construct was then transformed into *O. sativa* Indica rice through *Agrobacterium-*mediated transformation by using a protocol described by Toki et al. (2006). Briefly, 7-day-old scutellum-derived embryogenic calli were infected with *Agrobacterium* EHA105 strain harboring the above-mentioned construct. These calli were washed after three days of co-cultivation and kept on the selection medium supplemented with 50 μg/L hygromycin. The secondary calli that developed after four rounds of selection were then transferred to regeneration media for the development of shoots. The regenerated shoots were then transferred to the rooting media. The regenerated plantlets were maintained at 30 ± 2°C at a photosynthetic flux density of 300 μmol m^−2^s^−1^, 75–80% of humidity, and photoperiod duration of 16/10 h light/dark phase in rice growth medium (RGM) for 1 month. Thereafter, the plants were transferred to pots containing soil in the greenhouse having the same growth conditions as mentioned above.

### Heat Stress Treatment and Expression Profile of Marker Genes in *TaOBF1-5B* Overexpression Lines in *Arabidopsis thaliana* and *Oryza sativa*

For checking the basal thermotolerance in plants, the *Arabidopsis* Col-0 seeds and the overexpression line seeds were germinated on half strength MS media and allowed to grow in a culture room maintained at 22 ± 2°C at a photosynthetic flux density of 300 μmol m^−2^s^−1^, 60% of humidity and photoperiod duration of 16/10 h light/dark phase for 2 weeks. For basal heat stress, the plants were transferred to a growth chamber maintained at 42°C for 2 h and then returned to their original growth conditions for recovery. The phenotype was recorded after 5 days of recovery.

For checking the thermotolerance in rice, T_2_ seeds were germinated on MS medium supplemented with hygromycin. The WT seeds were germinated on only MS media. Five days after germination, the plants were transferred to a growth chamber maintained at 42°C at a photosynthetic flux density of 300 μmol m^−2^s^−1^, 60% of humidity, and a photoperiod duration of 16/10 h light/dark phase. After 5 days of heat stress, the plants were then transferred back to normal temperature and allowed to recover. The phenotype was observed after 4 days of recovery. For checking the expression of heat stress marker genes, plants were grown in the greenhouse until the heading stage and then were then subjected to alternate days of heat stress followed by recovery. The flag leaf tissue was harvested after the stress treatment and stored at −80°C until RNA isolation. For analyzing the ROS levels, 15-day-old seedlings were subjected to 42°C for 24 h followed by a single day of recovery after which both leaf and root tissues were used for NBT staining.

### Histochemical ROS Detection

For vizualization the ROS levels in *TaOBF1-5B Arabidopsis* overexpression lines and WT, staining with nitro blue tetrazolium (NBT) was done (Meena et al., 2020; Samtani et al., 2022). The seeds were germinated on half strength MS medium and then 1- and 2-week-old plants were subjected to 42°C for 2 h, after which the overnight staining of the seedlings was done with NBT (2 mM NBT powder, 20 mM phosphate buffer). The seedlings were washed with water and subjected to chlorophyll removal by dipping them in a bleaching solution (ethanol, acetic acid, and glycerol in a ratio of 3:1:1). The seedlings were then visualized under a bright field light microscope (Leica, Germany) and pictures were taken for comparison of ROS in transgenics and the wild-type plants.

Similarly, NBT staining was done in *TaOBF1-5B* rice overexpression lines and the WT plants after heat stress for analyzing the ROS levels. For this, 15-day-old seedlings grown hydroponically were subjected to 42°C for 24 h and then allowed to recover for 24 h after which the staining was done overnight as described above.

### Estimation of Chlorophyll Content and Fresh Weight

Two-week-old *TaOBF1-5B* transgenic and WT plants *A. thaliana* were subjected to 42°C for 2 h. For estimation of chlorophyll, 50 mg of the seedling tissue was taken in a test tube containing 2.5 ml of Dimethyl Sulfoxide and incubated overnight for chlorophyll bleaching. The absorbance of the samples was then measured at 645 nm and 663 nm in a UV-Vis spectrophotometer (Hitachi U-2810, Tokyo, Japan), and the chlorophyll content was calculated according to Arnon (1949); Agarwal and Khurana (2018). For fresh weight estimation, 2-week-old seedlings were subjected to the above-mentioned HS regime, and after 4 days of recovery, the fresh weight was measured. Three biological replicates having 50 seedlings each were taken for calculations.

### RNA Isolation and Quantitative Real-Time PCR Analysis

Total RNA was extracted using the TRIzol Reagent (Ambion) according to the manufacturer's protocol. Isolated RNA was converted into cDNA using the Applied Biosystems™ High-Capacity cDNA Reverse Transcription kit (Thermo Fisher Scientific). Quantitative Real-Time PCR (qRT-PCR) was conducted using the SYBR Green (Applied Biosystems) in QuantStudio™ 3 Real-time PCR system (Thermo Fisher Scientific) to study the expression of selected genes (primer sequences provided in Supplementary Table 1) with three biological and three technical replicates. For internal control, *GAPDH* was used in the case of *T. aestivum, Actin 2* in the case of *A. thaliana*, and *Ubiquitin5* was used in the case of *O. sativa*. Relative gene expression was calculated according to the 2–^ΔΔ^Ct method (Livak and Schmittgen, 2001). Further, the relative expression of *TaOBF1* homoeologs in different tissues was calculated according to Borah and Khurana (2018).

The real-time data presented in graphs depict the mean ± standard deviation of mean (SD). The distribution of the data was assumed to be normal and thus paired Student's t-test was used for statistical analyses of the results. The statistical significant differences were shown at *p* ≤ 0.05 (marked ^*^), *p* ≤ 0.01 (marked ^**^), and *p* ≤ 0.001 (marked ^***^).

## Results

### Identification and Phylogenetic Analysis of *TaOBF1-5B* and *TaOBF1-5D*

Genome-wide identification of *TabZIPs* in wheat showed the expression profile of all the bZIPs under heat stress conditions (Agarwal et al., 2019). In this data, Traes_5B_FF44610EF1.3 and Traes_5D_011851EE7.1 were identified as two *TabZIPs*, which showed upregulation by heat stress. Using the Ensembl database, these genes were found to be homoeologs and the domain analysis of the database identified them as *OBF1*. The Traes_5B_FF44610EF1.3 gene was found to be located on chromosome 5B (hereafter denoted as *TaOBF1-5B*) with CDS of 474 bp, protein of 157 amino acids, and 5' and 3' non-coding region of 598 and 372 bp, respectively. On the other hand, Traes_5D_011851EE7.1 was also found to be located on chromosome 5D (hereafter denoted as *TaOBF1-5D*) with CDS of 474 bp, protein of 157 amino acids, and 5' and 3' non-coding region of 320 and 295 bp, respectively. Sequence analysis revealed that the CDS of *TaOBF1-5B* and *TaOBF1-5D* differed only by the presence of eight SNPs as the rest of the sequence was found to be identical (Supplementary Figure S1A). At the protein level, differences of only two amino acids were found in these homoeologs. TaOBF1-5B had Ala and Asp, while in TaOBF1-5D Ser and Thr were present at respective positions (Supplementary Figure S1B). By using the differences in their UTR regions, the CDS of *TaOBF1-5B* and *TaOBF1-5D* was cloned into the pENTR/D-TOPO vector and subsequently mobilized into various destination vectors (Supplementary Figure S2).

For finding the evolutionary links of TaOBF1, a phylogenetic tree was constructed using OBF and TGA sequences from different species (Supplementary Figure S1C). Interestingly, all the OBFs appeared in a separate clade, whereas all the TGAs grouped in different clades. This grouping pattern indicates their functional divergence as well. Moreover, TaOBF1-5B and TaOBF1-5D were found to be closest to OBF1 of *Secale cereale* (Supplementary Figure S1C).

### Comparison of Expression Profile and Subcellular Localization of TaOBF1-5B and TaOBF1-5D

A wheat expression database was used to investigate the differences in the expression profile of *TaOBF1-5B* and *TaOBF1-5D*. Supplementary Figure S3 depicts the expression of *TaOBF1-5B* and *TaOBF1-5D* across various Zadok stages and in different developmental tissues. It was observed that both of them had similar expression profiles, as among different Zadok stages their expression was found in stem (Zadok stage 65) and root (Zadokstage 13 and Zadokstage 23) (Supplementary Figure S3A). Amongst the various tissues, *TaOBF1-5B* and *TaOBF1-5D* showed the highest expression in the stamen, although it was found to be higher for *TaOBF1-5B* (Supplementary Figure S3B). The stress responsiveness of *TaOBF1* was also analyzed by the wheat expression database (Supplementary Figure S3C). Both *TaOBF1-5B* and *TaOBF1-5D* were found to be upregulated by 6 h of heat stress.

**Figure 1:**
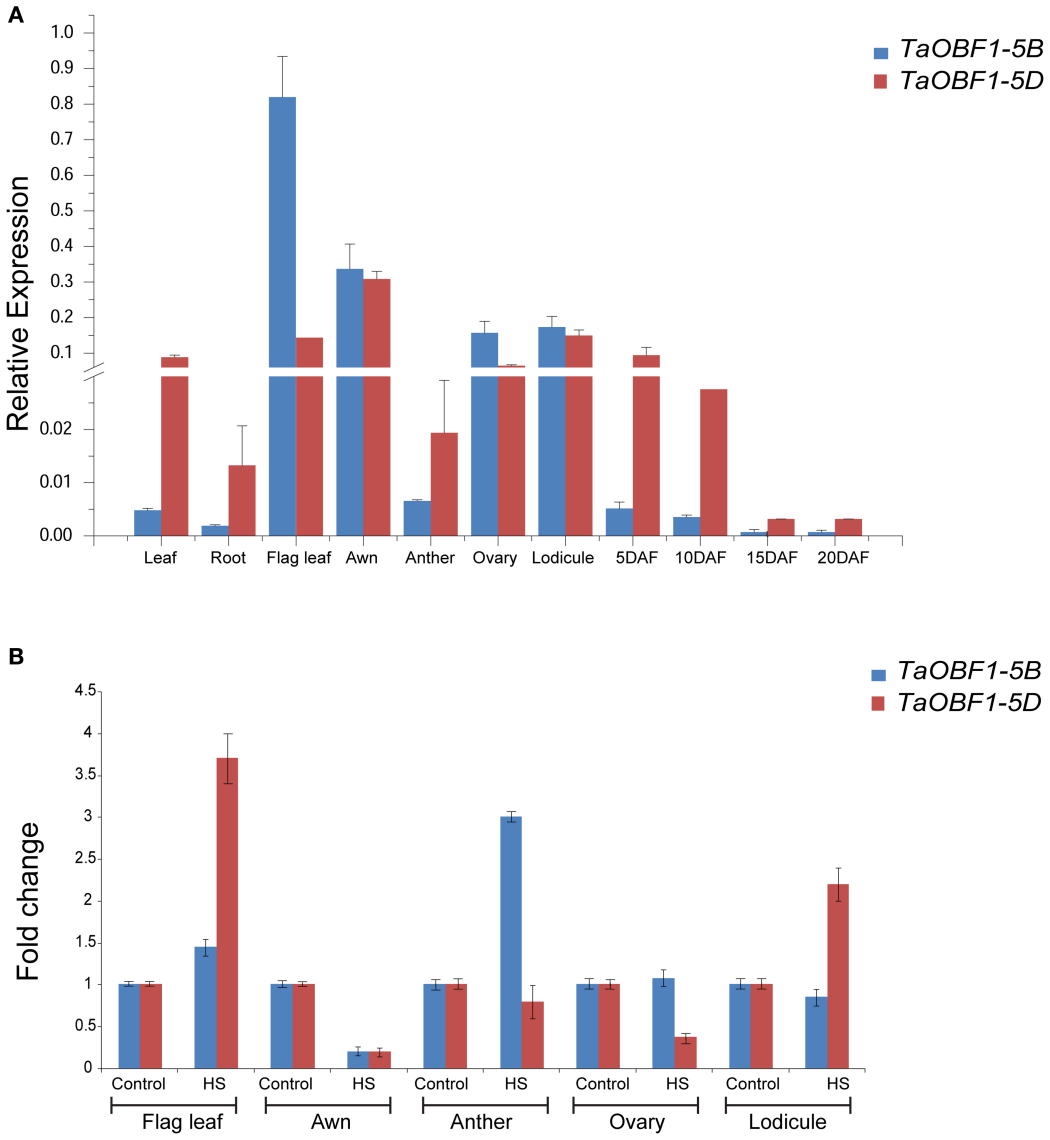
Expression analysis of TaOBF1 homoeologs in different tissues of wheat cv. PBW343 seedlings under **(A)** control and **(B)** heat stress conditions. Relative expression was checked in different tissues by qRT-PCR. *TaGAPDH* was used as an internal control gene. Error bars indicate values ±SD. DAF, days after fertilization.

**Figure 2:**
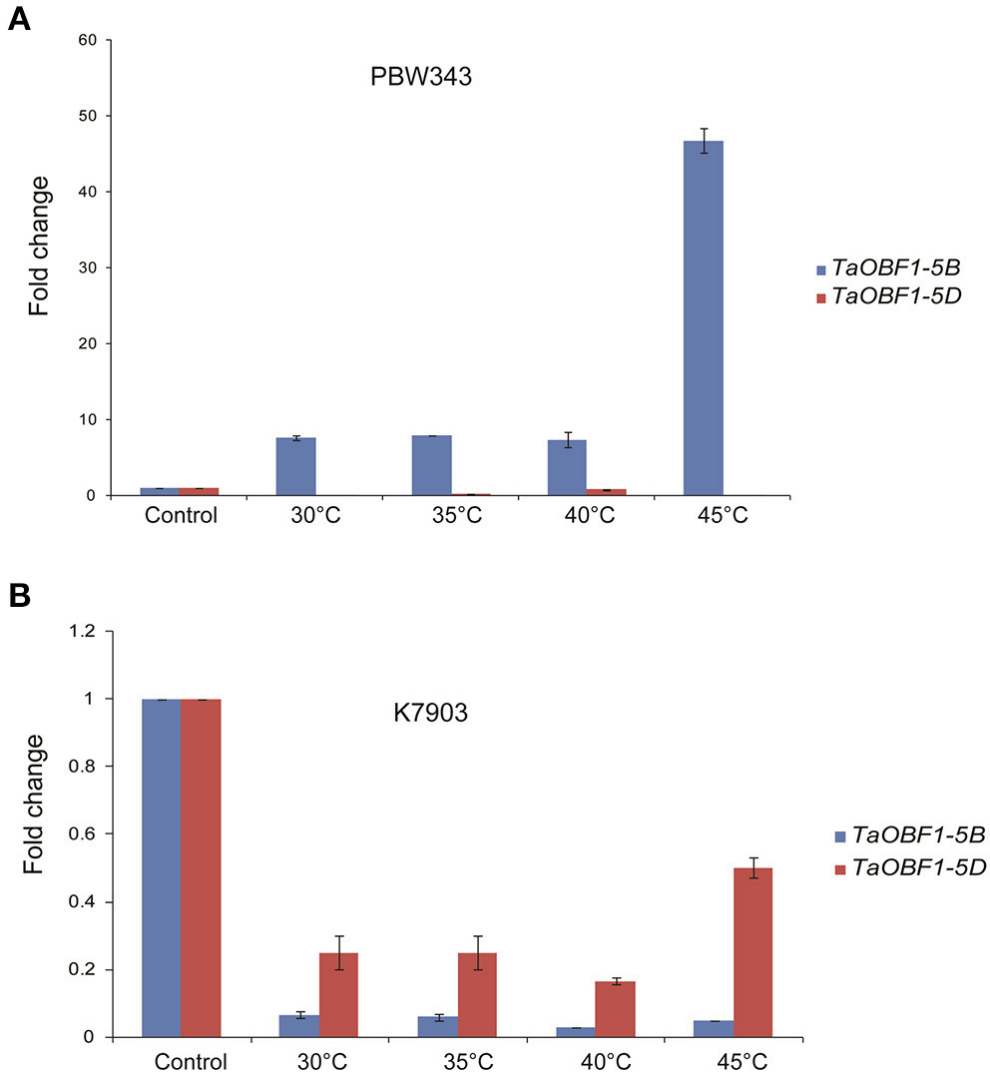
Expression analysis of *TaOBF1* homoeologs under heat stress conditions in *Triticum aestivum* cv. **(A)** PBW343 (heat sensitive) **(B)** K7903 (heat tolerant) seedlings at various temperatures. Relative fold change was checked by qRT-PCR. *TaGAPDH* was used as an internal control gene. Error bars indicate values ± SD.

**Figure 3:**
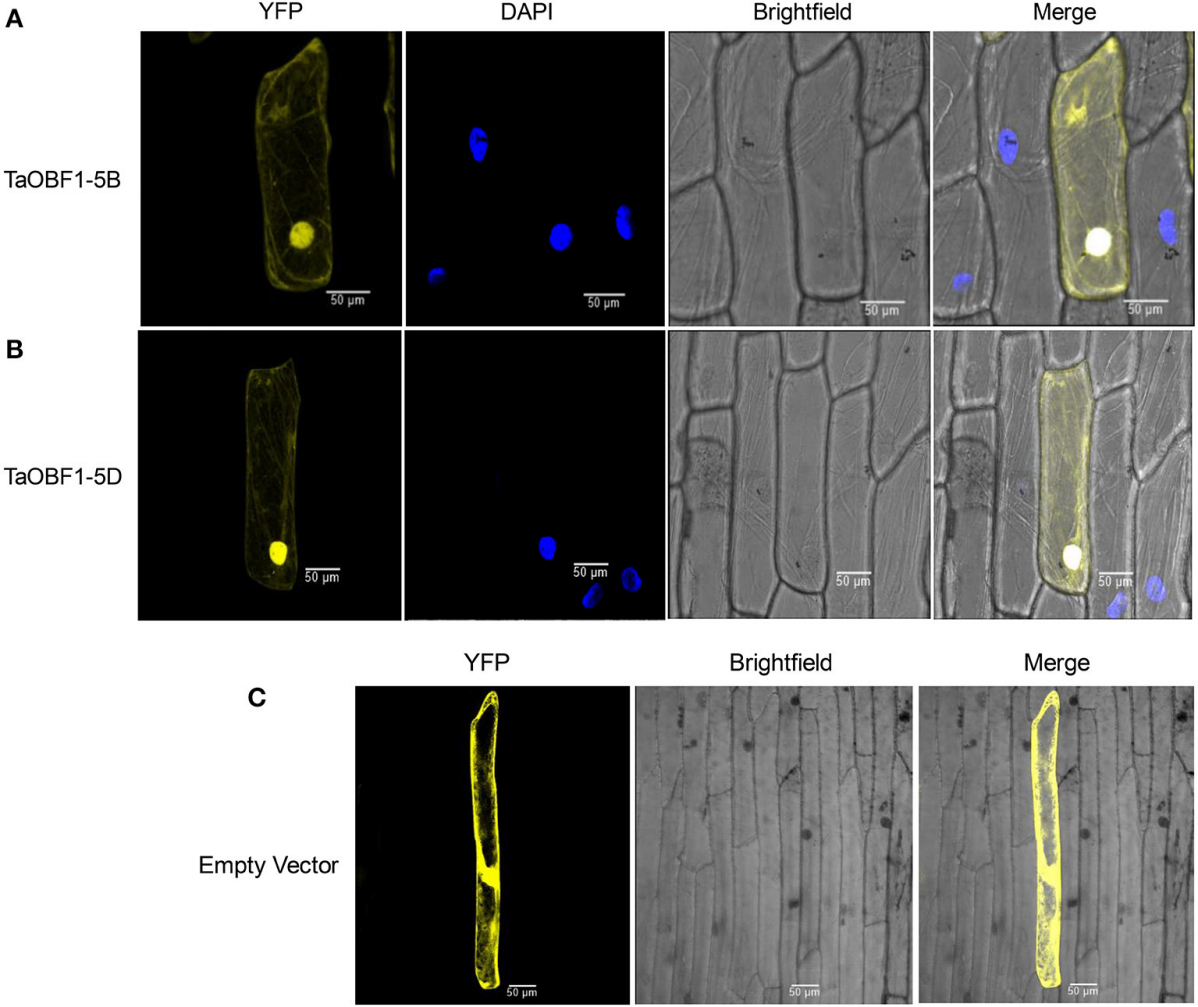
Subcellular localization of selected TaOBF1 homoeologs **(A)** TaOBF1-5B, **(B)** TaOBF1-5D, and **(C)** localization of pSIT3CA empty vector. DAPI stain was used to highlight the nucleus along with the YFP (YFP, yellow fluorescent protein) (scale bar 50 μm).

Further, the relative expression pattern of both the homoeologs of *TaOBF1* was analyzed in different tissues in Indian cultivars, i.e., PBW 343 by RT-PCR (Figure 1A). Interestingly, as compared to the online expression data of the cultivar Chinese Spring, a differential expression pattern of *TaOBF1* homoeologs, was observed in PBW-343. *TaOBF1-5B* had higher expression levels in flag leaf, awn, and lodicule tissues as compared to *TaOBF-5D*. Moreover, their expression was also compared in control and under heat stress conditions in flag leaf, awn, anther, ovary, and lodicule tissues (Figure 1B). *TaOBF1-5D* was found to be heat-induced in the flag leaf and lodicule tissues, whereas *TaOBF1-5B* was observed to be specifically upregulated by the heat stress in the anther tissues. Their expression was also validated at different time points of heat stress in the Indian wheat cultivar PBW-343. As shown in Supplementary Figure S4, *TaOBF1-5B* was induced by heat stress within 30 mins and its expression peaked at 4 h. Surprisingly, *TaOBF1-5D* was not found to be upregulated by heat stress. The expression *TaOBF1-5B* and *5D* were also compared in heat-sensitive (PBW-343) and heat tolerant (K7903) wheat varieties at different temperatures (Figure 2). Higher transcript levels of *TaOBF1-5B* were observed with increasing temperatures in the heat-sensitive variety, i.e., PBW-343, with its highest expression at 45°C (Figure 2A). The expression of *TaOBF1-5D* did not show any increase with the rise in temperature in PBW-343. In the case of heat-tolerant wheat varieties, the expression of both the homoeologs of TaOBF1 were found to be down-regulated with the increase in temperature (Figure 2B). However, lesser down-regulation of *TaOBF1-5B* was observed as compared to *TaOBF1-5D*. Since a differential expression pattern was noticed for *TaOBF1* homoeologs, therefore, their subcellular localization pattern was also checked by bombarding their YFP-fusion constructs in onion epidermal peel cells. Both TaOBF1-5B and TaOBF1-5D were found to localize to the nucleus and in the cytoplasm (Figure 3). No difference in their localization pattern was observed. Thus, these studies indicate that among the TaOBF1 homoeologs, *TaOBF1-5B* has a probable role in the heat stress response, and thus it was taken further for in-depth characterization.

### Homo and Hetro-Dimerization Between TaOBF1-5B and TaOBF1-5D

**Figure 4:**
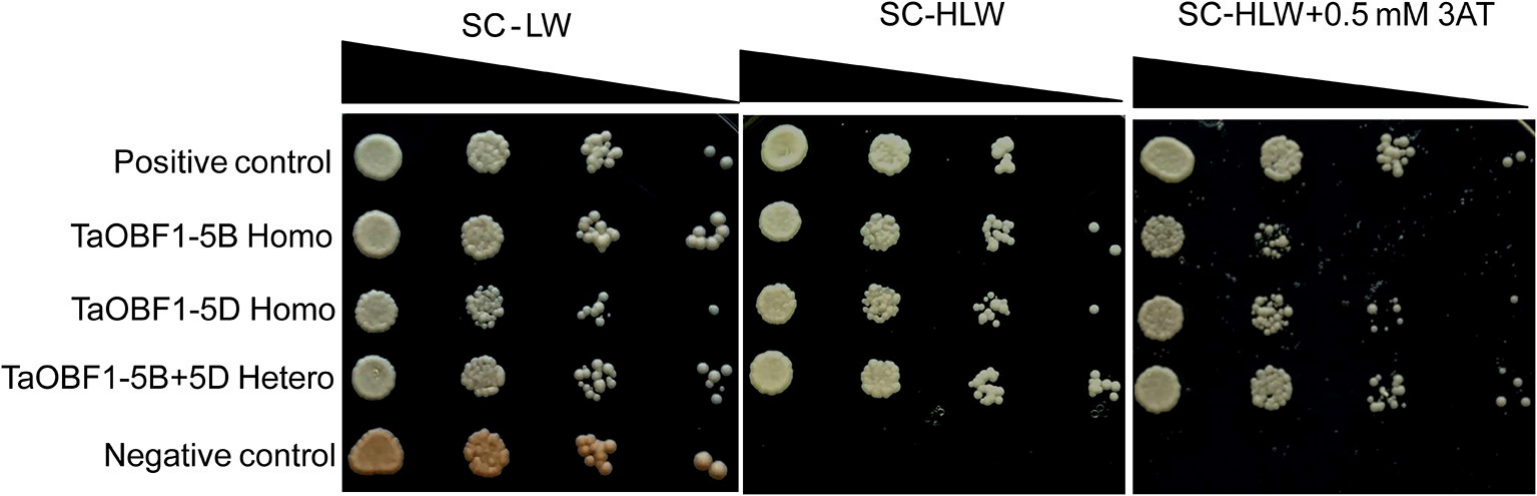
Homo-dimerization and hetero-dimerization of TaOBF1-5B and TaOBF1-5D. Yeast-two-hybrid (Y2H) assay was performed to check the homodimerization of TaOBF1-5B and TaOBF1-5D by co-transforming pGBKT7-5B and pGADT7-5B (labeled as TaOBF1-5B Homo) and pGBKT7-5D and pGADT7-5D (labeled as TaOBF1-5D Homo), respectively. The Y2H assay was performed to check the heterodimerization of TaOBF1-5B and TaOBF1-5D by co-transforming pGBKT7-5B and pGADT7-5D (labeled as TaOBF1-5B-5D Hetero). The growth of co-transformed yeast cells was analyzed by drop assay on SD/-Leu/-Trp (-LW) medium, SD/-Leu/-Trp/-His (-HLW) medium, and SD/-Leu/-Trp/-His (-HLW) + 0.5 mM 3-aminotriazole medium (3AT). Pair of plasmids pGBKT7-53 and pGADT7-T were used as positive control, and pGBKT7-Lam and pGADT7-T were used as the negative control.

As *TaOBF1* belongs to a bZIP family, which is well known for controlling the transcription of various genes. Therefore, the transactivation potential of TaOBF1-5B was checked by a yeast one-hybrid system. As shown in Supplementary Figure S5, no growth of the transformed cells was observed on histidine lacking media thereby indicating the absence of transactivation. The leucine zipper motif of bZIPs is known to dimerize (Jakoby et al., 2002). Therefore, the homo and hetero-dimerization of both the homoeologs of TaOBF1 were checked using the yeast two-hybrid assay. To see the interaction, the growth of co-transformed colonies was analyzed on SC-HLW and SC-HLW + 0.5 mM 3AT media. Homo-dimerization was observed with both TaOBF1-5B and TaOBF1-5D (Figure 4). TaOBF1-5D showed slightly better strength of homodimerization as compared to TaOBF1-5B as observed by their growth on SC-HLW + 0.5 mM 3AT media. Apart from this, TaOBF1-5B and TaOBF1-5D were also found to hetero-dimerize as the co-transformed cells were able to grow on 3AT-containing media. The strength of hetero-dimerization was found to be more as compared to the individual homo-dimerization.

### *In vivo* Interaction of TaOBF1-5B With TaSTI and TaHSP90

**Figure 5:**
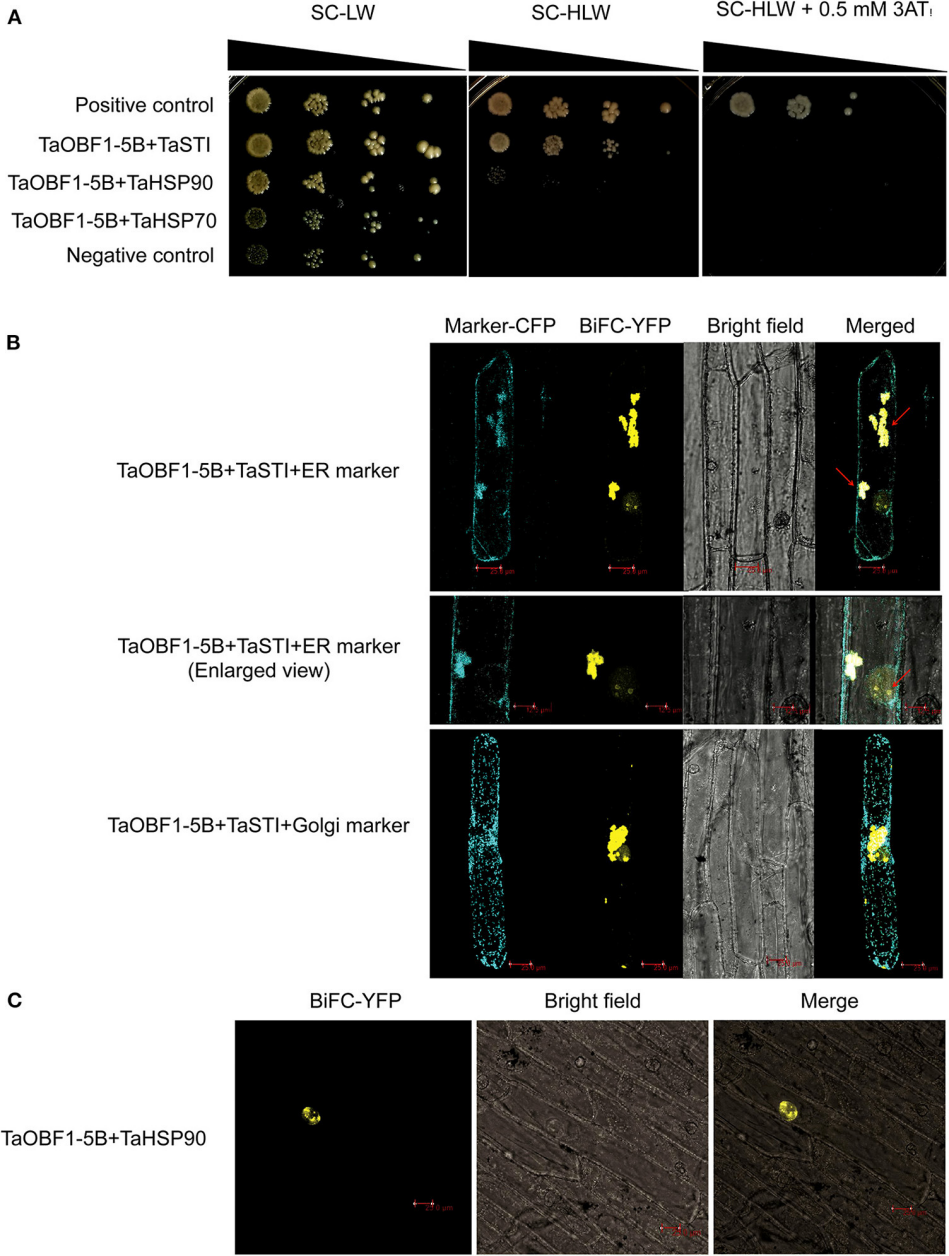
Interaction of TaOBF1-5B with TaSTI and TaHSP90. **(A)** The yeast-two-hybrid assay was performed to check the interaction of TaOBF1-5B with TaSTI, TaHSP90, and TaHSP70 proteins. The growth of co-transformed yeast cells was analyzed on SD/-Leu/-Trp (-LW) medium and on SD/-Leu/-Trp/-His (-HLW) medium. Drop assay was performed on media lacking histidine, leucine, and tryptophan (-HLW) along with 0.5 mM 3-aminotriazole (3AT). Pair of plasmids pGBKT7-53 and pGADT7-T were used as positive control, and pGBKT7-Lam and pGADT7-T were used as the negative control. **(B)** BiFC assay showing the interaction of TaOBF1-5B with TaSTI-2A in the nucleolus and ER in onion epidermal cells. The localization of the BiFC signal was comparable with the ER marker localization. Red arrows indicate the interaction occurring in the ER and in the nucleolus [ER (endoplasmic reticulum); Scale bar 25 μm]. **(C)** BiFC assay showing the interaction of TaOBF1-5B with TaHSP-90 in the nucleus in onion epidermal cells (scale bar 25 μm).

Shimizu et al. (2005) reported that the low-temperature induced Lip19 protein interacted with OsOBF1 to work in the cold-signaling pathway. As *TaOBF1-5B* was found heat-responsive, therefore, we checked its interaction with TaSTI, which is a stress-induced protein known to function in heat stress. Also, previously we had reported the interaction between TaSTI and TaHSP90 (Meena et al., 2020), therefore, we also checked the interaction of TaOBF1-5B with TaHSP90 and TaHSP70 using the yeast two-hybrid method. Interestingly, TaOBF1-5B was found to interact both with TaHSP90 and with TaSTI as the co-transformed yeast cells were able to grow in an SC-HLW medium (Figure 5A). Interaction with TaSTI appeared to be stronger in comparison to TaHSP90. However, no interaction was observed with TaHSP70. These interactions were further confirmed by the BiFC assay. Interaction between TaOBF1-5B and TaSTI was found to occur in the nucleolus and the ER (Figure 5B). On the other hand, the interaction between TaOBF1-5B and TaHSP90 was seen only in the nucleus (Figure 5C).

### TaOBF1-5B Enhances Basal Thermotolerance in *Arabidopsis thaliana*

**Figure 6:**
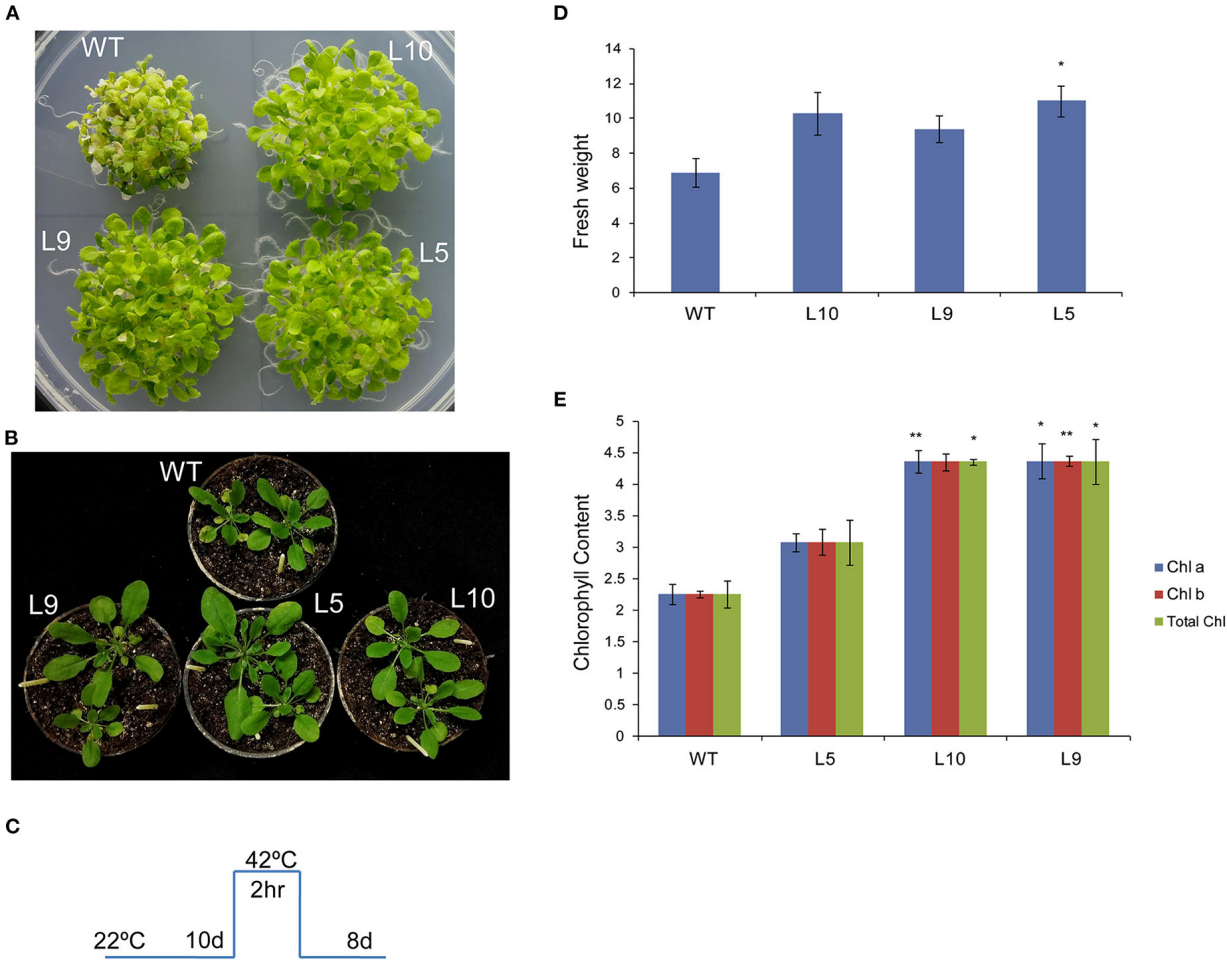
Overexpression of *TaOBF1-5B* in Arabidopsis *thaliana* promoted better growth and more chlorophyll levels under basal heat stress conditions. **(A,B)** Photographs showing thermotolerance in *Arabidopsis* TaOBF1-5B-overexpression lines. **(C)** Depiction of stress regime. **(D,E)** Graphs representing fresh weight chlorophyll content, respectively. Asterisks indicates statistically significant difference. (Student *t*-test: *p ≤ 0.05; **p ≤ 0.01).

**Figure 7:**
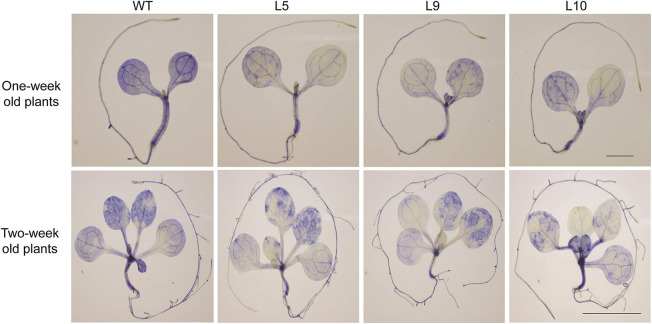
NBT staining of 1 week (scale bar 1 mm) and 2 weeks (scale bar 1 cm) old seedlings. Plants were given heat stress and were then stained with NBT to visualize the superoxide anion accumulation after the stress treatment.

To investigate the role of TaOBF1-5B in heat stress response, it was overexpressed in the *A. thaliana* plants. The overexpression lines were confirmed by using hygromycin-specific PCR, gene-specific PCR (Supplementary Figures S6A,B). The level of ectopic overexpression was detected by RT-PCR, and the overexpression lines were found to have higher expression in comparison to the WT (Supplementary Figure S6C). For checking the basal thermotolerance, *Arabidopsis* seedlings were subjected to heat stress of 42°C for 2 h and then allowed to recover for 8 days (Figure 6C). The overexpression lines were able to recover better as observed by lesser senescence, more fresh weight, and more chlorophyll levels in comparison to the wild-type plants (Figures 6A,D,E). Similarly, when the 1-week-old plants were subjected to heat stress in pots, they grew better than wild-type plants, as observed by their rosettes (Figure 6B). It is well reported that heat stress accelerates ROS accumulation in plants, which leads to oxidative damage (Suzuki and Mittler, 2006). Therefore, the levels of ROS accumulation were checked in both WT and over-expression lines after giving heat stress at two different stages (Figure 7). The overexpression lines had lesser ROS levels in comparison to WT as evident by the NBT staining.

### Transcription Profiling of Stress Marker Genes in *Arabidopsis*

#### TaOBF1-5B Overexpression Lines

**Figure 8:**
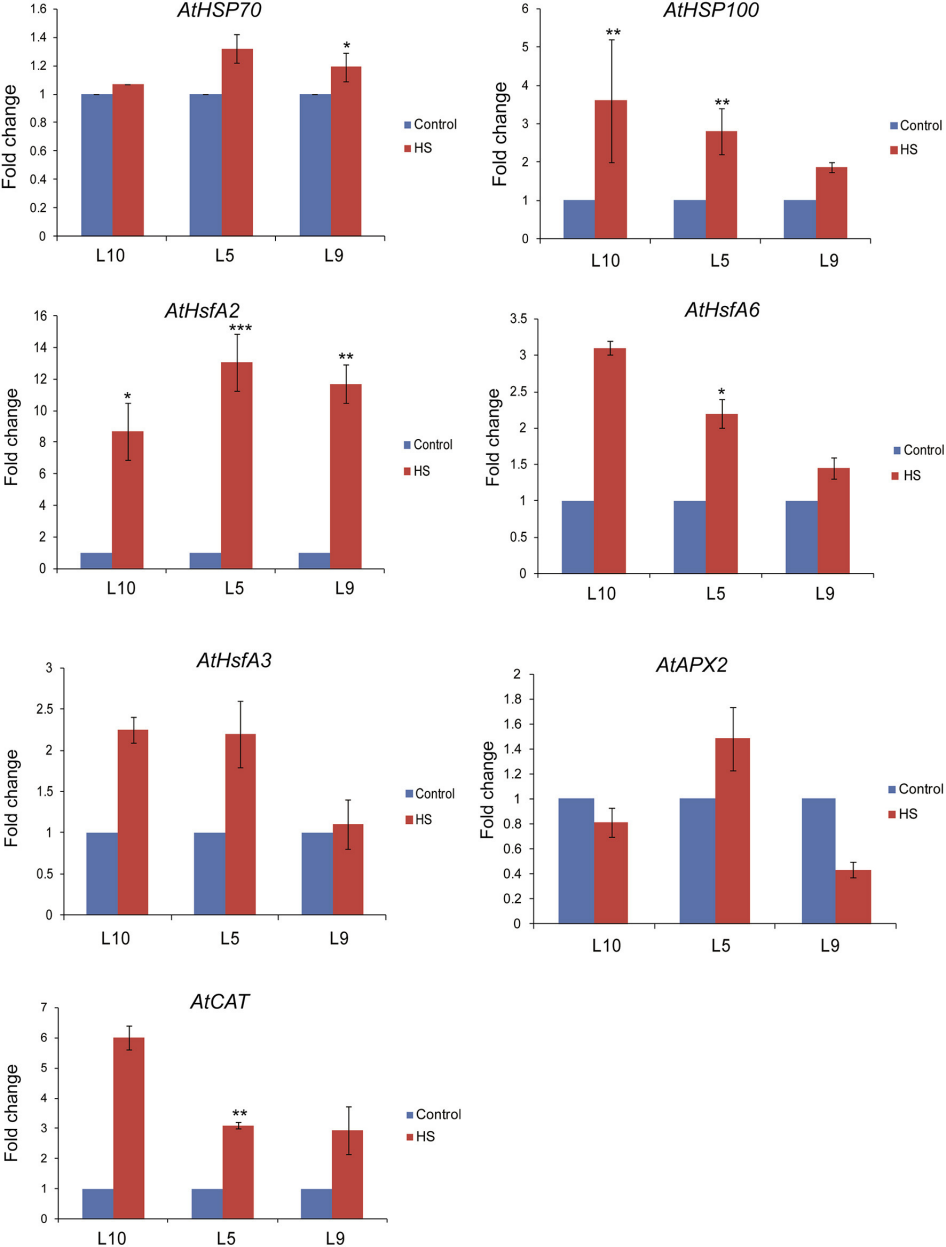
Expression analysis of heat stress marker genes by qRT-PCR of WT and *Arabidopsis* transgenics under control and heat stress. Transcript levels were normalized to WT and *AtActin* was used as an internal control. Error bars indicate values ± SD. Asterisks on top of the error bars represent the significance levels (Students *t*-test; *p* ≤ 0.05).

To understand the molecular basis of *TaOBF1-5B* mediated heat stress tolerance in *A. thaliana*, we checked the expression levels of different *Hsfs* and *HSPs*, which are major components of HSR. It was observed that relative expression levels of *AtHsfA2, AtHsfA6*, and *AtHsfA3* were higher in the overexpression lines as compared to the WT under heat stress conditions (Figure 8). Moreover, an increase in the expression of *AtHSP100* was also observed in overexpression lines under stress conditions in comparison to WT. However, no difference in *AtHSP70* levels was observed between the overexpression and the WT plants. As the overexpression lines showed lower ROS accumulation under heat stress conditions, therefore, the expression levels of antioxidant enzymes, such as *Ascorbate peroxidase 2 (APX2)* and *Catalase (CAT)*, were also analyzed. All the overexpression lines had higher levels of *AtCAT* as compared to WT (Figure 8). No significant difference was observed in the case of *AtAPX2*.

### Analysis of *TaOBF1-5B* Rice Transgenics Showed Lesser ROS Accumulation Under Heat Stress Conditions

After observing the thermotolerance in *A. thaliana, TaOBF1-5B* was then overexpressed in a monocot model organism, i.e., *O. sativa*, for confirming its role in providing thermotolerance. *Agrobacterium*-mediated transformation method was used for transforming rice calli with *TaOBF1-5B* and the regenerated plants were selected on hygromycin-containing media (Supplementary Figure S7). The transgenic lines were confirmed by using hygromycin specific PCR, and gene-specific PCR (Supplementary Figures S8A,B). The level of ectopic overexpression was detected by RT-PCR and high expression levels were detected in the overexpression lines 2, 8, and 7 with relative transcript levels of 175, 140, and 150, respectively, as compared to 1 in WT (Supplementary Figure S8C). Thus, these three independent transgenic lines were selected for further analysis.

**Figure 9:**
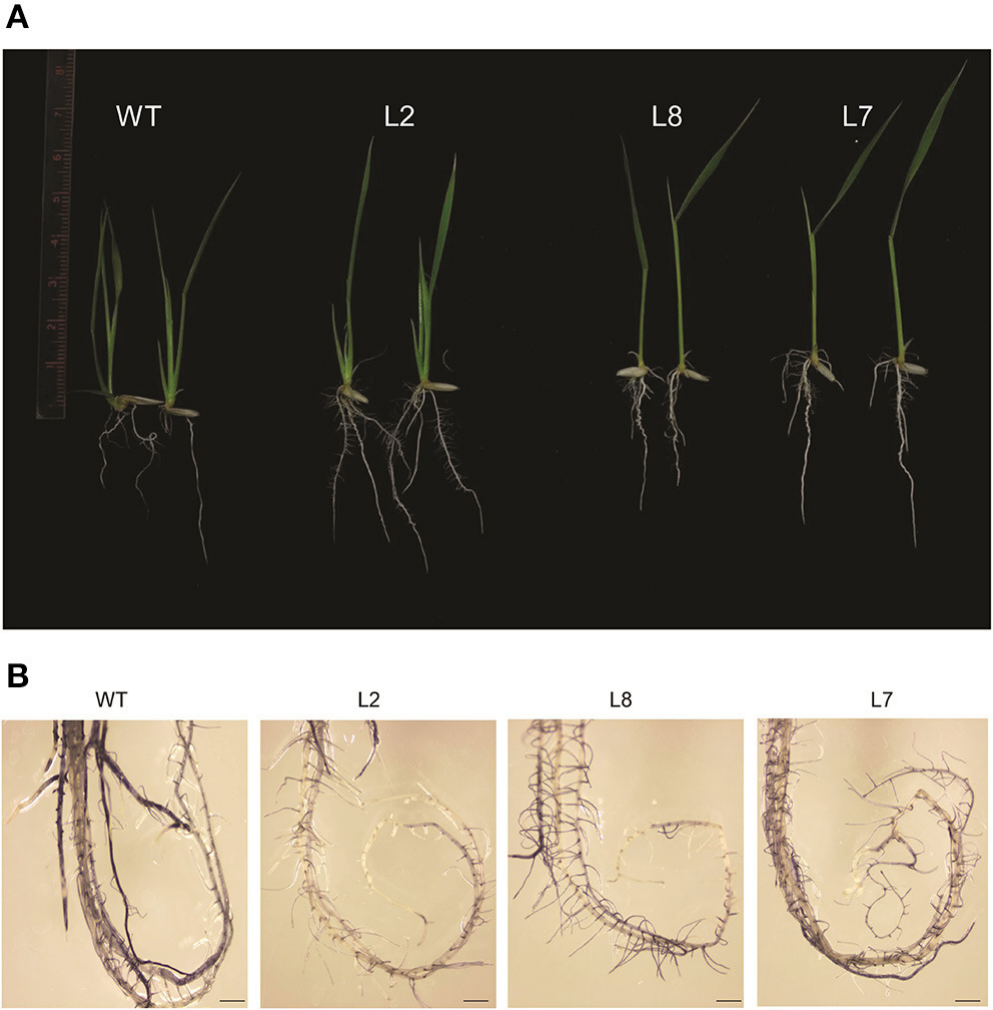
Phenotypic analysis of rice *TaOBF1-5B-*overexpression lines under heat stress conditions. **(A)** Photographs showing thermotolerance in TaOBF1-5B rice overexpression lines. Transgenic plants showed better growth as compared to WT. **(B)** NBT staining of roots of WT and *TaOBF1-5B* transgenics. Plants subjected to heat stress followed by the recovery were stained with NBT to analyze the superoxide anion accumulation after the stress treatment (scale bar 3 mm).

For checking the thermotolerance in the selected over-expression lines of rice, the 5-day-old seedlings were subjected to 42°C for 5 days and then allowed to recover. The *TaOBF1-5B* overexpression lines were seen to perform better than the WT (Figure 9A). The transgenic plants showed better shoot and root growth, while the growth of WT halted during heat stress. Apart from this, the level of the oxidative load was also analyzed in the transgenic plants after the heat stress. For this, 15-day-old seedlings were subjected to 42°C for 24 h followed by a single day of recovery after which both leaf and root tissues were used for NBT staining. Although no significant difference was observed among the leaf tissues, *TaOBF1-5B* overexpression lines showed lesser ROS accumulation in the roots as compared to the wild-type plants (Figure 9B). This indicated the potential role of *TaOBF1-5B* in providing thermotolerance.

### Transcript Profiling of Stress Marker Genes in Rice *TaOBF1-5B* Overexpression Lines

**Figure 10:**
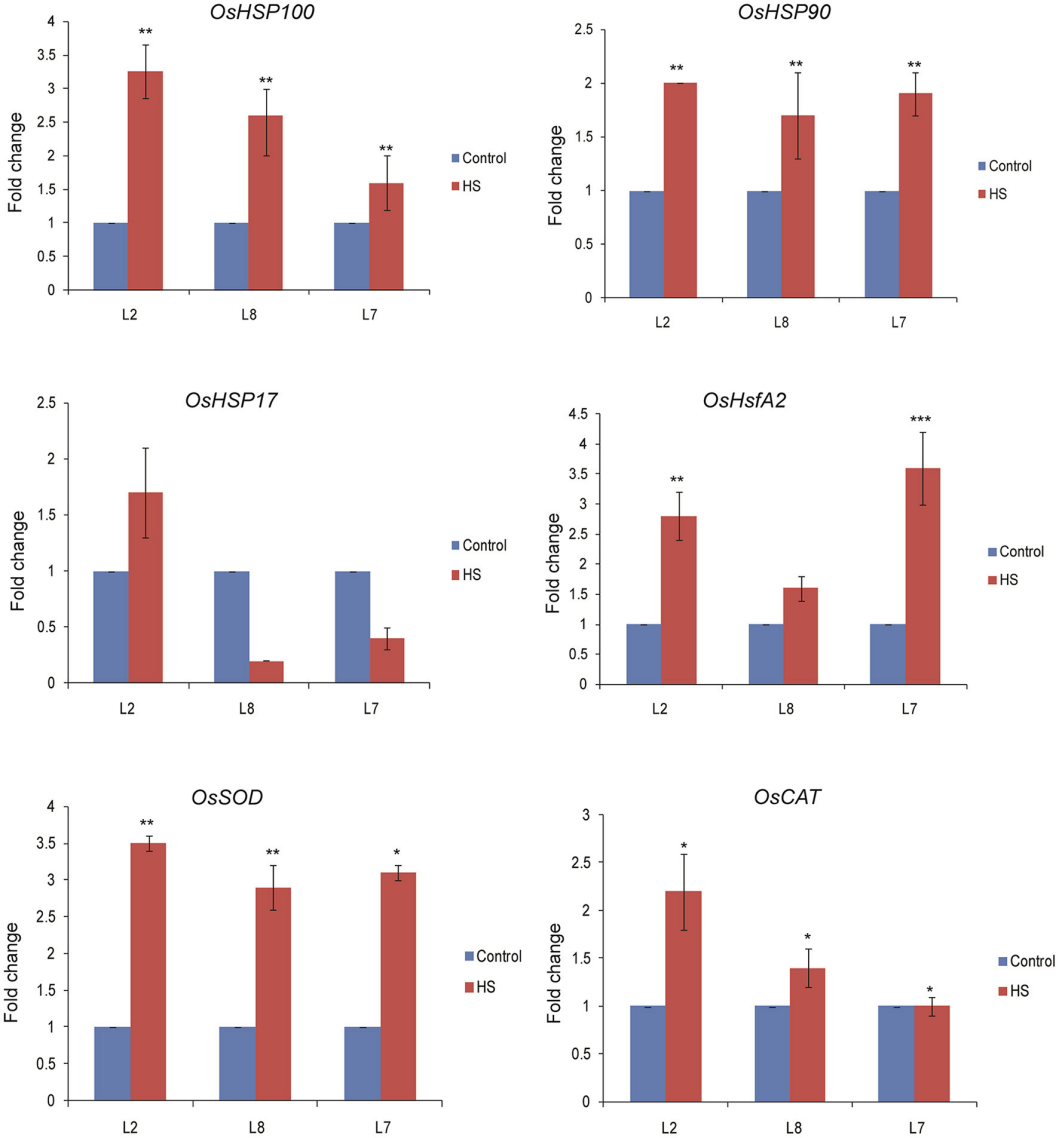
Expression analysis of heat stress marker genes by qRT-PCR of WT and *TaOBF1-5B rice* transgenics under control and heat stress. Transcript levels were normalized to WT and *OsUbiquitin* was used as an internal control. Error bars indicate values ± SD. Asterisks on top of the error bars represent the significance levels (Students *t*-test; *p* ≤ 0.05).

To explore the mechanism behind *TaOBF1-5B* attributed thermotolerance in rice, we analyzed the expression levels of stress marker genes in *TaOBF1-5B* overexpression lines. As observed in Figure 10, higher transcript levels of *OsHSP100 and OsHsfA2* were observed in the transgenic plants in comparison to WT under heat stress conditions. A slight but significant increase was observed in the case of *OsHSP90* levels in transgenics, whereas no difference was observed in the levels of OsHSP17. Apart from these, the expression levels of antioxidant enzymes, such as *Superoxide dismutase* (*OsSOD*) and *Catalase* (*OsCAT*), were also analyzed. Higher expression levels of *OsSOD* were detected in the overexpression lines as compared to WT after exposure to heat stress conditions. No significant difference in the expression of *OsCAT* was observed between the transgenic and WT plants (Figure 10).

## Discussion

bZIP transcription factors have been well documented to work in various abiotic stresses, such as drought, salinity, and cold stress (Nijhawan et al., 2008; Sornaraj et al., 2016). However, till now there have been limited reports regarding the role of bZIPs in heat stress response in plants. Previously, Agarwal et al. (2019) identified *bZIPs* in *T. aestivum* and had reported the role of one of the TabZIP (Traes_7AL_25850F96F.1) in providing thermotolerance in *A. thaliana*. Using this genome-wide data, we have identified *TaOBF1* as one of the most heat-responsive bZIP present in *T. aestivum*. Moreover, both the homoeologs of *TaOBF1* (*TaOBF1-5B* and *TaOBF1-5D*) appeared to be heat-responsive in this data. Therefore, in this study, we tried to find the functional role of *TaOBF1* during the heat stress response in plants.

Wheat being a hexaploid plant has three sub-genomes, which leads to the presence of homoeologs of each gene. Although it has been speculated that there may be partitioned expression patterns among the homoeologous genes, the experimental evidence is still limited (Zhang et al., 2016). To elucidate the differences between *TaOBF1* homoeologous, their CDS sequence and protein sequences were analyzed. It was observed that at the gene level they differ by 8 SNPs whereas at the protein level a change in two amino acids was seen (Supplementary Figure S1). The change of Ala to Ser and Asp to Thr was found between *TaOBF1-5B* and *TaOBF1-5D*. Ser and Thr residues have been documented as the potential targets of phosphorylation in bZIPs (Smykowski et al., 2015). Therefore, it can be speculated that TaOBF1-5D might work after being phosphorylated and activated by a kinase. Both TaOBF1-5B and TaOBF1-5D showed localization in the nucleus and the cytoplasm, which indicates that they might work in these compartments (Figure 3).

The available online expression database suggested a similar expression pattern of both the homeologues of *TaOBF1* under heat stress conditions. However, a significant difference in their expression pattern was noticed after running RT-PCR for the Indian cultivar PBW-343 (Figure 2A). This observed difference could be explained by the fact that the data present in the wheat expression database is from the Chinese Spring variety. Moreover, *TaOBF1-5B* and *TaOBF1-5D* showed differential expression in various tissues under control and heat stress conditions (Figure 1). This indicates their tissue preferential expression pattern, as the D homoeologs of TaOBF1 might function in flag leaf and lodicule under heat stress, whereas the B homoeologs might function in the anthers. A recent report by Meena et al. (2022) showed that the homoeologs of TaHsfA6b showed differential expression patterns in various tissues under control conditions. Apart from the comparison in tissues, their expression profiling under heat stress conditions in the seedling stage revealed *TaOBF1-5B* as a heat-inducible gene. *TaOBF1-5D* was not found to be heat inducible at the seedling stage. Moreover, in heat-tolerant cultivar its expression was found to be severely down-regulated (Figure 2B). Thus, overall, these studies hint toward the expression partitioning of *TaOBF1* homoeologs in response to heat stress. This could be further supported by the observations by Liu et al. (2015) and Meena et al. (2020), wherein differences in homoeologs expression have been observed under heat stress conditions for other genes.

Since TaOBF1-5B was chosen for further analysis, its transcriptional activity was checked and it was found that TaOBF1-5B lacked the transactivation potential (Supplementary Figure S5). Previous studies have reported that OsOBF1 and TaOBF1 work by interacting with another bZIP i.e. lip19 (Shimizu et al., 2005; Kobayashi et al., 2008). So, it can be speculated that TaOBF1 might regulate its target genes *via* interaction with other bZIPs. Interestingly, TaOBF1-5B was found to interact with TaSTI and TaHSP90, which are known to work in heat stress response (Figure 5). This was found to be in accordance with earlier reports, which have highlighted the role of OBF1 in cold-signaling in rice and abiotic stress tolerance in wheat through its interaction with lip19 protein (Shimizu et al., 2005; Kobayashi et al., 2008).

Overexpression of *TaOBF1-5B* enhanced the thermal stress tolerance of *A. thaliana*, as the plants recovered better after heat stress as compared to the WT (Figures 6, 7). Similarly, in the case of rice, the transgenic lines showed thermotolerance and accumulated lesser superoxide ions after heat stress (Figure 9). Thus, this data suggested that *TaOBF1-5B* might have a role in providing basal thermal tolerance. In support of this, the expression levels of various stress marker genes, such as *AtHsfA2, AtHSfA6, AtHSfA3, AtHSP100*, and *AtCat*, were found to be higher in overexpression lines of *A. thaliana* (Figure 8). On the other hand, levels of *OsHsfA2, OsHSP100, OsSOD*, and *OsHSP90* were observed to be higher in rice transgenics after exposure to heat stress (Figure 10). The role of these marker genes in attributing to thermotolerance has been reported earlier (Jacob et al., 2017; Samtani et al., 2022). Also, it is worth noticing that overexpression of *TaSTI* and *TaHSP90* has been documented to promote thermotolerance and *TaSTI* overexpression lines of *Arabidopsis* had higher levels of *AtHsfA2* and *AtHsfA6* transcripts under heat stress conditions (Vishwakarma et al., 2018; Meena et al., 2020). In this study as well, expression levels of *AtHsfA2* and *AtHsfA6* were found to be high in overexpression lines of *Arabidopsis* under heat stress conditions. So, this highlights the probability of TaOBF1 working in concert with *TaSTI* and *TaHSP90* in the HSR to promote thermotolerance.

Apart from this, we had previously shown that TaSTI interacts with TaHSP90 in the nucleus and the ER-Golgi complex (Meena et al., 2020). Since TaOBF1-5B was found to interact with both of these proteins, we speculated that three of them might work in a complex. However, the BiFC assay showed that TaSTI and TaOBF1-5B interacted in the nucleolus and the ER, whereas interaction with TaHSP90 was observed in the nucleus (Figures 5B,C). Moreover, ER retention signals have been documented in TaSTI (Meena et al., 2020). Thus, this ruled out the possibility of the three working together. Therefore, it is possible that TaOBF1-5B by interacting with TaSTI moves in the ER and might function in the protein unfolding response. Apart from this, it is well known that HSP90 binds to Hsfs and regulates their activity (Yamada and Nishimura, 2008). As TaOBF1-5B interacted with TaHSP90 in the nucleus, it could be possible that TaHSP90 might regulate its activity, though further experimental evidence is needed to confirm this.

**Figure 11:**
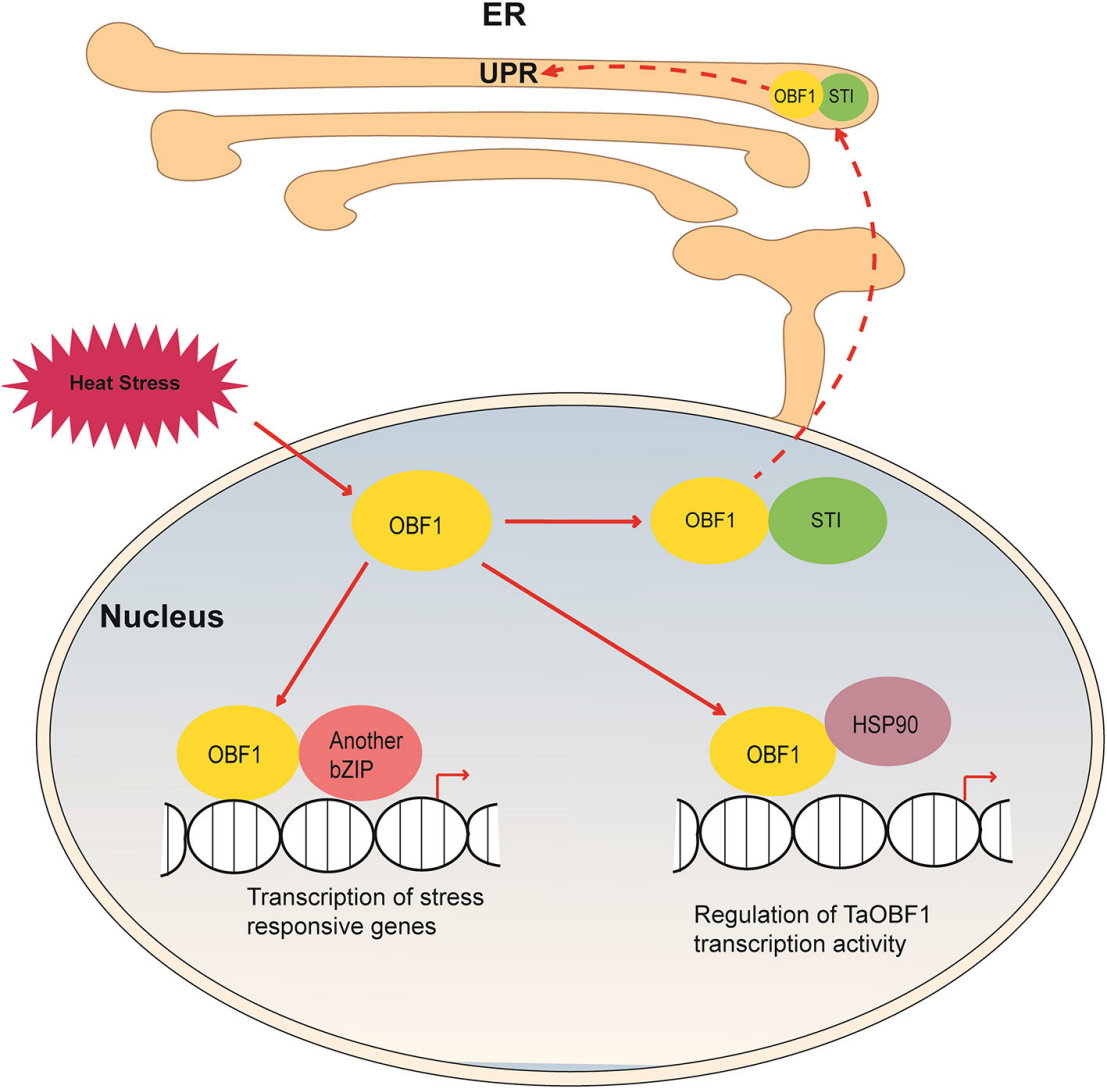
Hypothetical model depicting the role of TaOBF1 in heat stress response. Upon heat stress, TaOBF1 by interacting with TaHSP90 translocates from nucleus to the ER where it participates in the protein unfolding response. Also inside the nucleus, it forms homodimers and heterodimers (by interaction with other bZIPs) and regulates the transcription of heat stress-responsive genes, which aid in providing thermotolerance. Apart from this, TaHSP90 also interacts with TaOBF1 and regulates its transcriptional activity.

Based on our findings, we propose a hypothetical model depicting the role of TaOBF1 in response to high temperatures (Figure 11). Upon heat stress, TaOBF1-5B on interacting with TaSTI translocates into the ER, where it might participate in protein unfolding response. Apart from this, homo-dimerization of TaOBF1-5B or hetero-dimerization with some other bZIP transcription factor leads to the transcription of heat stress-responsive genes, which help in imparting thermotolerance. Its interaction with TaHSP90 probably regulates its transcriptional activity. However, the molecular mechanisms behind TaHSP90 mediated regulation need further investigation. Also, how these three types of interactions help in providing thermotolerance is a potential area of future research. Overall, this study identifies TaOBF1-5B as a positive regulator of heat stress tolerance and provides insights into the mechanisms involved in the heat stress response pathway in crops, such as wheat.

## Data Availability Statement

The raw data supporting the conclusions of this article will be made available by the authors, without undue reservation.

## Author Contributions

PK conceived the idea, concept of the research work, and provided all the facilities for the experiments. HS and AS performed the experiments and wrote the manuscript. All authors have read and agreed to the submitted version of the manuscript.

## Funding

HS and AS are thankful to the University Grant Commission (UGC) for the research fellowship. This work has been supported by a grant from the JC Bose Fellowship Award and Science and Engineering Research Board, Government of India.

## Conflict of Interest

The authors declare that the research was conducted in the absence of any commercial or financial relationships that could be construed as a potential conflict of interest. The handling editor declared a shared affiliation with the author PK at the time of the review.

## Publisher's Note

All claims expressed in this article are solely those of the authors and do not necessarily represent those of their affiliated organizations, or those of the publisher, the editors and the reviewers. Any product that may be evaluated in this article, or claim that may be made by its manufacturer, is not guaranteed or endorsed by the publisher.

## Supplementary Material

Supplementary Figure 1 Sequence and phylogenetic analysis of TaOBF1. **(A)** Sequence alignment of CDS of *TaOBF1-5B* and *TaOBF1-5D*. SNPs are depicted by #. **(B)** Protein sequence alignment of TaOBF1-5B and TaOBF1-5D.The difference in amino acids is depicted in yellow color. **(C)** Phylogenetic tree of TaOBF1 protein and OBF1 protein from other plant species. The unrooted tree was made using MEGA (version 7) with the neighbor joining method (1,000 replicates).

Supplementary Figure 2 Cloning of *TaOBF1* homeologues in different vectors. **(A)** Confirmation of *TaOBF1-5B* cloned in pDRIVE, pENTR/D-TOPO, and pMDC-32 vectors by digestion. **(B)** Confirmation of *TaOBF1-5D* cloned in pDRIVE, pENTR/D-TOPO, and pMDC-32 vectors by digestion.

Supplementary Figure 3 Expression analysis of TaOBF1 homoeologs in different developmental stages of wheat. **(A)** Heatmap showing the relative expression profile of the *TaOBF1-5B* and *TaOBF1-5D* across the various zadok stages of wheat. **(B)** Heatmap showing the relative expression profile of the *TaOBF1-5B* and *TaOBF1-5D* across the various tissues of wheat. **(C)** Heatmap showing the relative expression profile of the *TaOBF1-5B* and *TaOBF1-5D* under abiotic stresses and biotic stresses. MI, mock inoculation; HS, heat stress; DS, drought stress; CS, cold stress; FGI, *Fusarium graminearum* inoculation; PEG, polyethylene glycol.

Supplementary Figure 4 Expression analysis of TaOBF1 homoeologs in wheat cv. PBW343 seedlings at different time points of heat stress conditions. Relative fold change was checked by qRT-PCR. *TaGAPDH* was used as an internal control gene. Error bars indicate values ± SD.

Supplementary Figure 5 Transcriptional activation assay of TaOBF1-5B in yeast cells. TaOBF1-5B lacked transcriptional activation, as AH109 yeast cells containing TaOBF1-5B::pGBKT7 construct were unable to grow on SC-HW media.

Supplementary Figure 6 Confirmation of *TaOBF1-5B Arabidopsis* overexpression transgenic lines by **(A)** Hygromycin resistance gene specific PCR **(B)** Gene-specific PCR **(C)** RT-PCR.

Supplementary Figure 7 Generation of rice transgenics by tissue culture. Transformation of rice calli with *Agrobacterium* harboring *TaOBF1-5B* gene.

Supplementary Figure 8 Confirmation of *TaOBF1-5B* overexpression rice transgenic lines by **(A)** Hygromycin resistance gene specific PCR **(B)** Gene and vector-specific PCR **(C)** RT-PCR.

Supplementary Table 1 List of primers used in this study.
