# Supplementary figures and images for: Wheat ocs-Element Binding Factor 1 Enhances Thermotolerance by Modulating the Heat Stress Response Pathway
Source: Front Plant Sci. 2022 May 31;13:914363. doi: 10.3389/fpls.2022.914363 (PMC9194769; doi:10.3389/fpls.2022.914363)

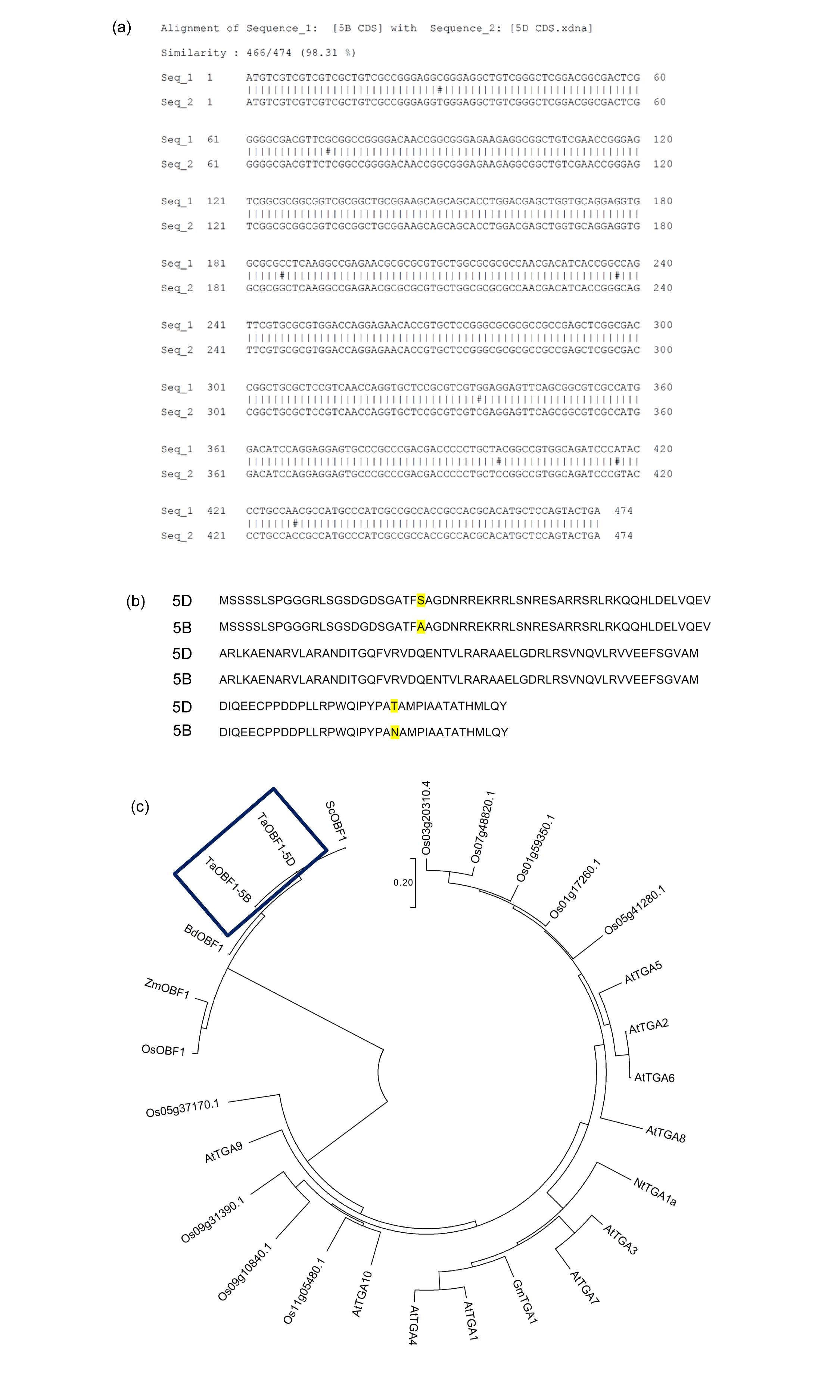

Supplement: Supplementary Figure 1 — Sequence and phylogenetic analysis of TaOBF1. (A) Sequence alignment of CDS of TaOBF1-5B and TaOBF1-5D. SNPs are depicted by #. (B) Protein sequence alignment of TaOBF1-5B and TaOBF1-5D.The difference in amino acids is depicted in yellow color. (C) Phylogenetic tree of TaOBF1 protein and OBF1 protein from other plant species. The unrooted tree was made using MEGA (version 7) with the neighbor joining method (1,000 replicates). [file Image_1.TIF]

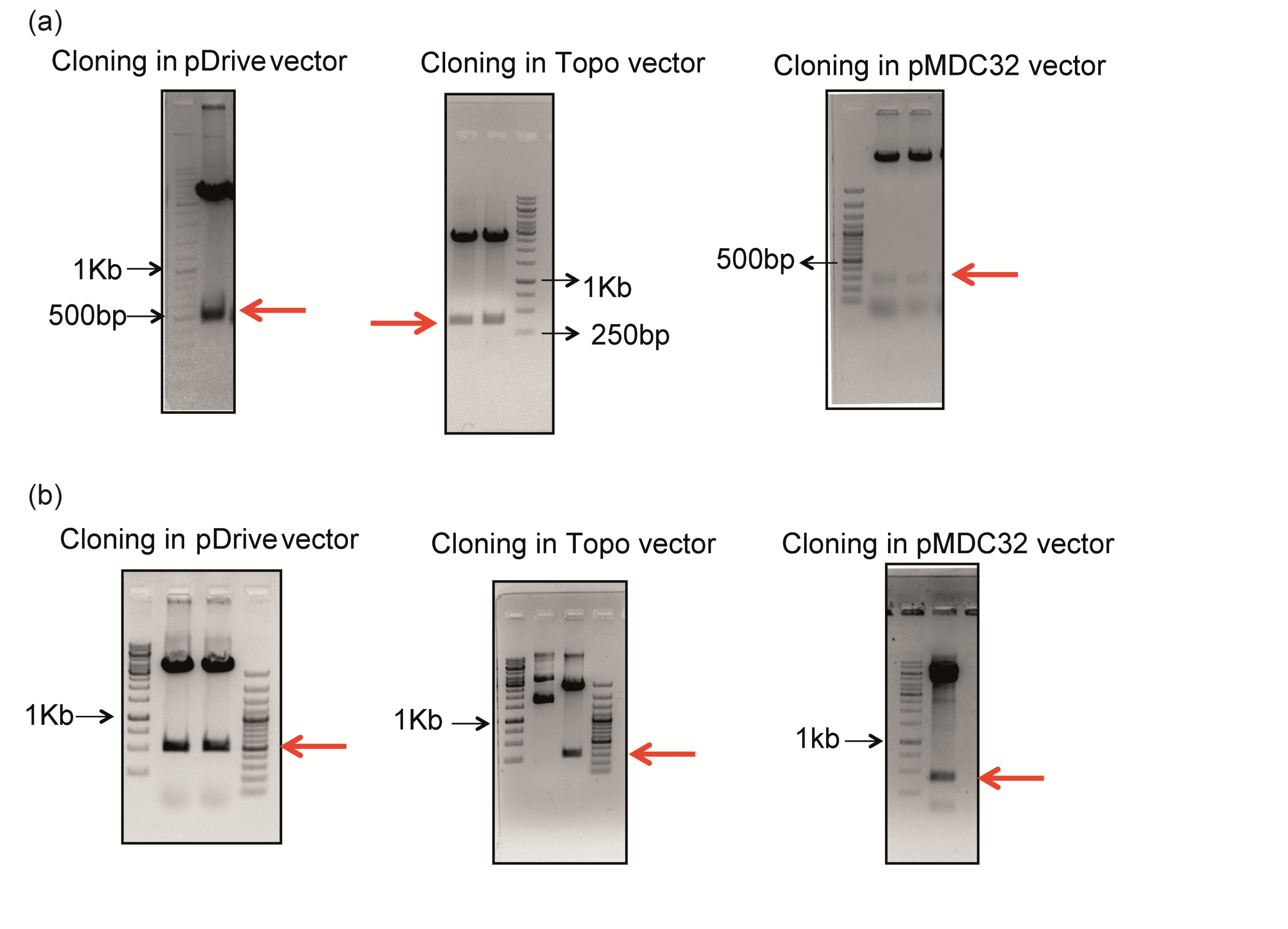

Supplement: Supplementary Figure 2 — Cloning of TaOBF1 homeologues in different vectors. (A) Confirmation of TaOBF1-5B cloned in pDRIVE, pENTR/D-TOPO, and pMDC-32 vectors by digestion. (B) Confirmation of TaOBF1-5D cloned in pDRIVE, pENTR/D-TOPO, and pMDC-32 vectors by digestion. [file Image_2.TIF]

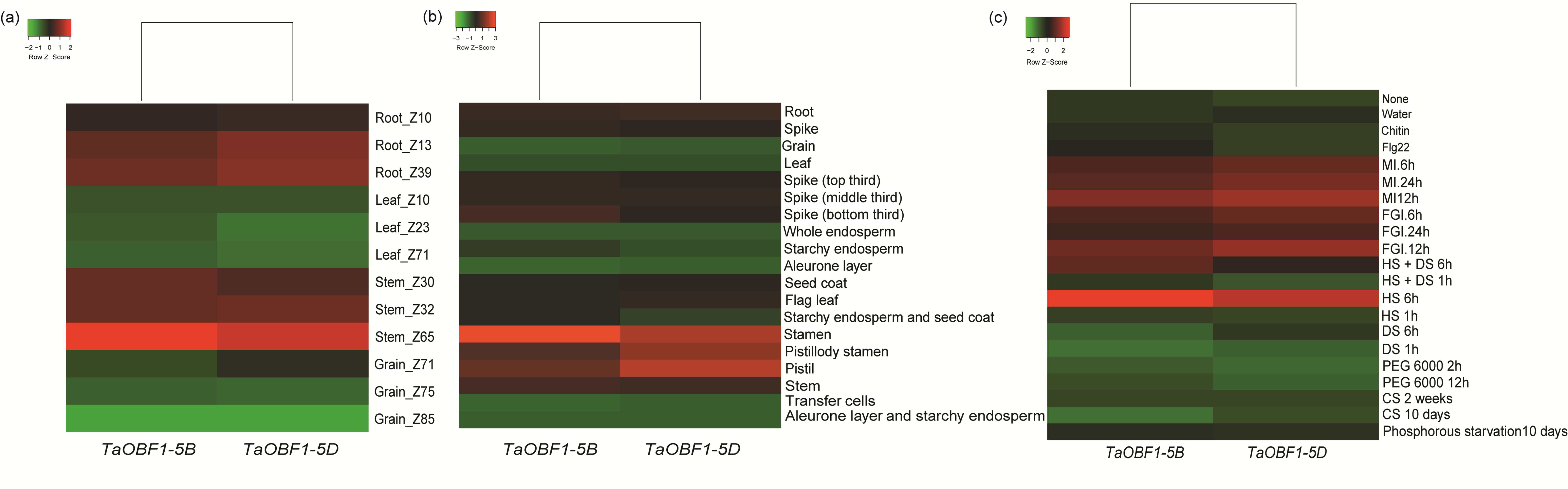

Supplement: Supplementary Figure 3 — Expression analysis of TaOBF1 homoeologs in different developmental stages of wheat. (A) Heatmap showing the relative expression profile of the TaOBF1-5B and TaOBF1-5D across the various zadok stages of wheat. (B) Heatmap showing the relative expression profile of the TaOBF1-5B and TaOBF1-5D across the various tissues of wheat. (C) Heatmap showing the relative expression profile of the TaOBF1-5B and TaOBF1-5D under abiotic stresses and biotic stresses. MI, mock inoculation; HS, heat stress; DS, drought stress; CS, cold stress; FGI, Fusarium graminearum inoculation; PEG, polyethylene glycol. [file Image_3.TIF]

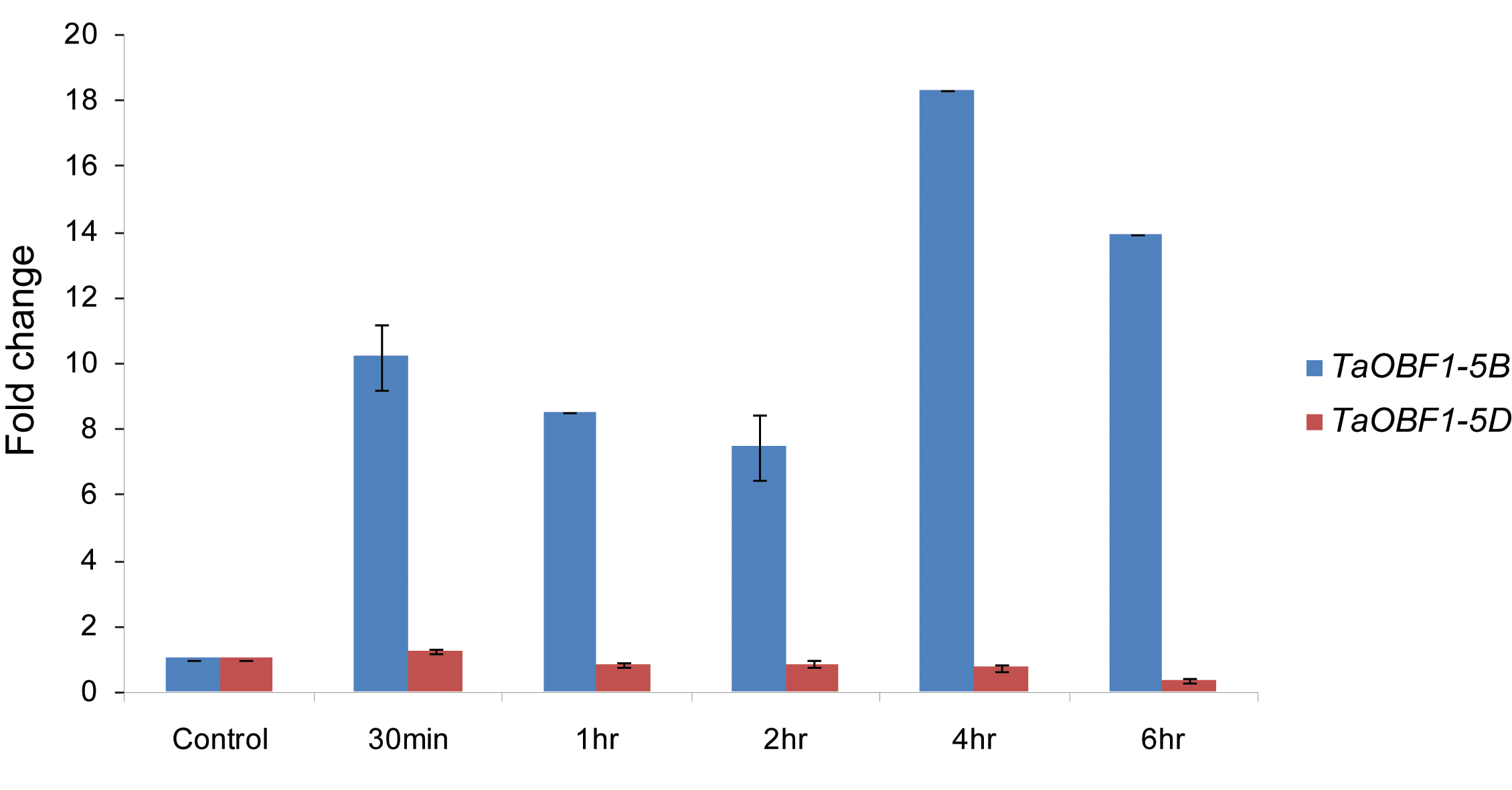

Supplement: Supplementary Figure 4 — Expression analysis of TaOBF1 homoeologs in wheat cv. PBW343 seedlings at different time points of heat stress conditions. Relative fold change was checked by qRT-PCR. TaGAPDH was used as an internal control gene. Error bars indicate values ± SD. [file Image_4.TIF]

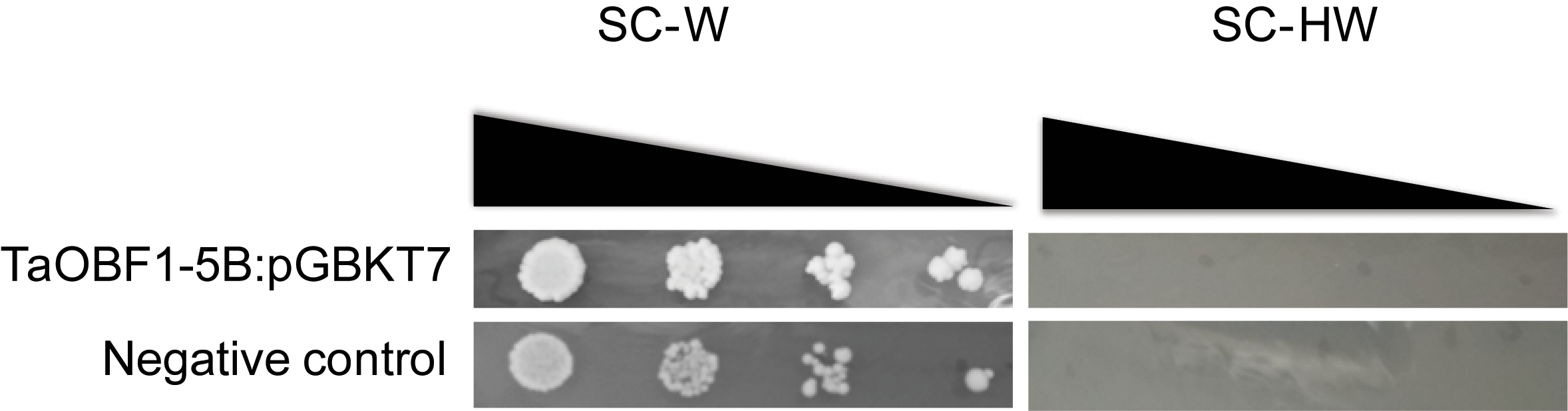

Supplement: Supplementary Figure 5 — Transcriptional activation assay of TaOBF1-5B in yeast cells. TaOBF1-5B lacked transcriptional activation, as AH109 yeast cells containing TaOBF1-5B::pGBKT7 construct were unable to grow on SC-HW media. [file Image_5.TIF]

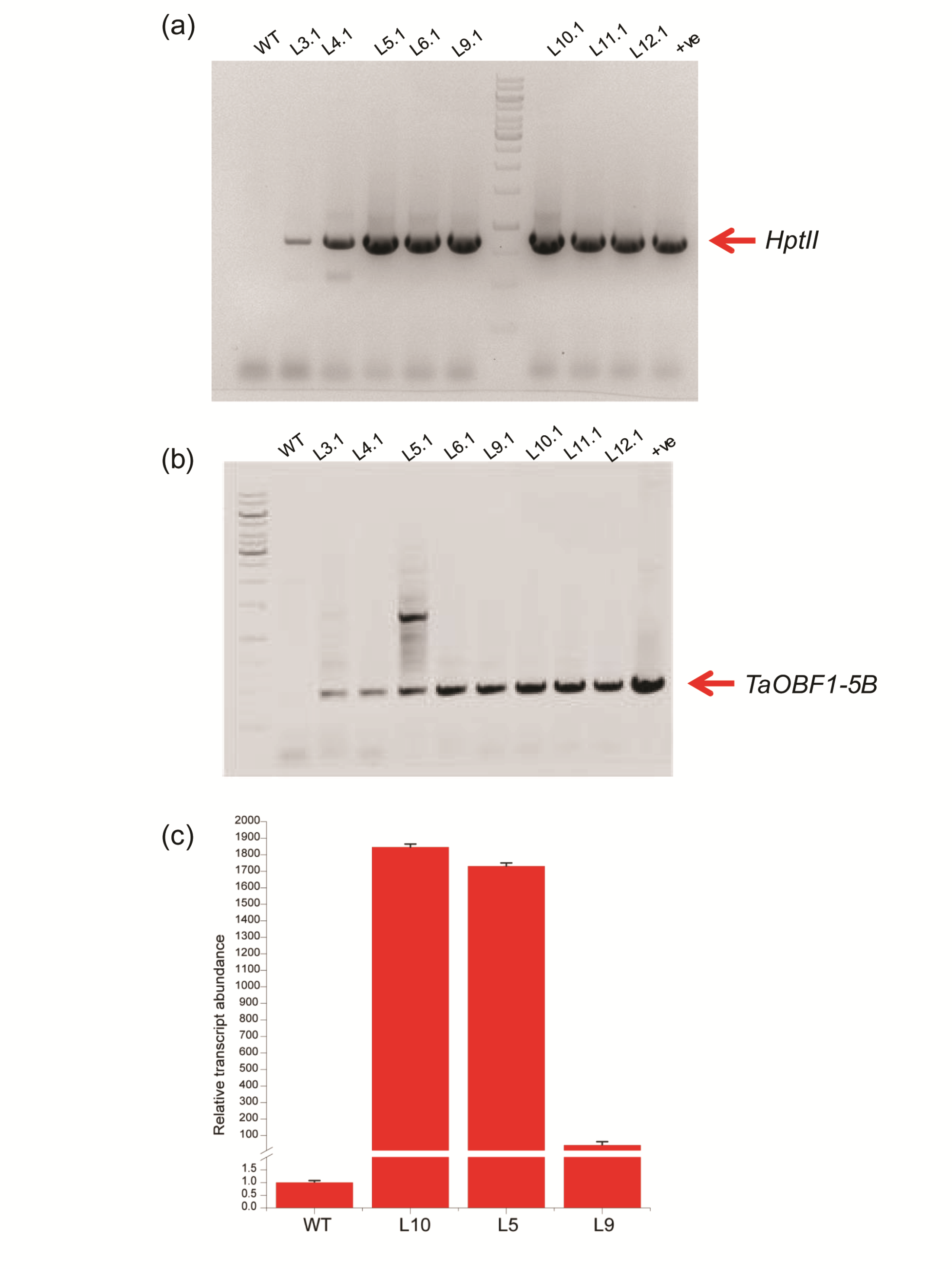

Supplement: Supplementary Figure 6 — Confirmation of TaOBF1-5B Arabidopsis overexpression transgenic lines by (A) Hygromycin resistance gene specific PCR (B) Gene-specific PCR (C) RT-PCR. [file Image_6.TIF]

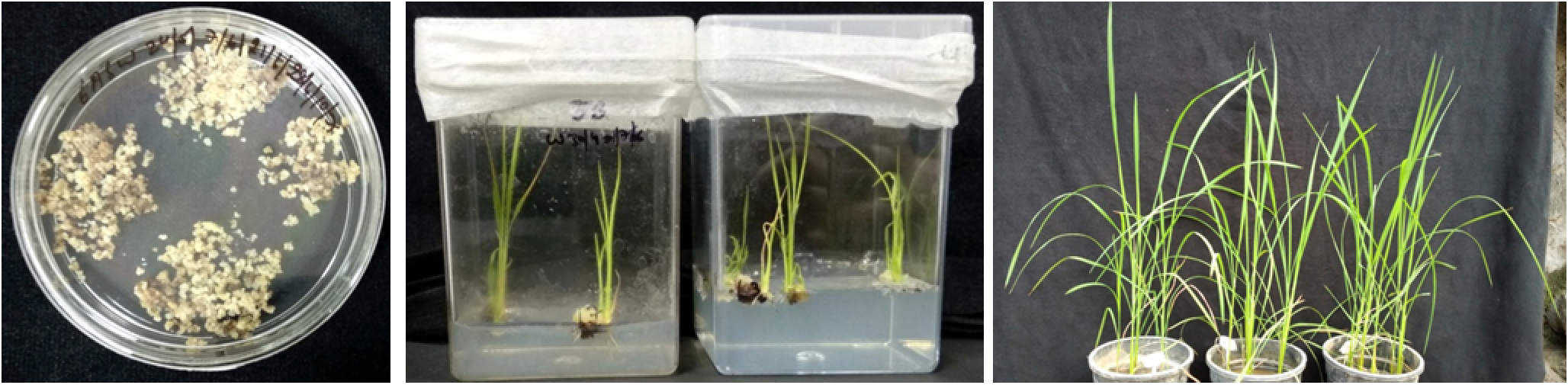

Supplement: Supplementary Figure 7 — Generation of rice transgenics by tissue culture. Transformation of rice calli with Agrobacterium harboring TaOBF1-5B gene. [file Image_7.TIF]

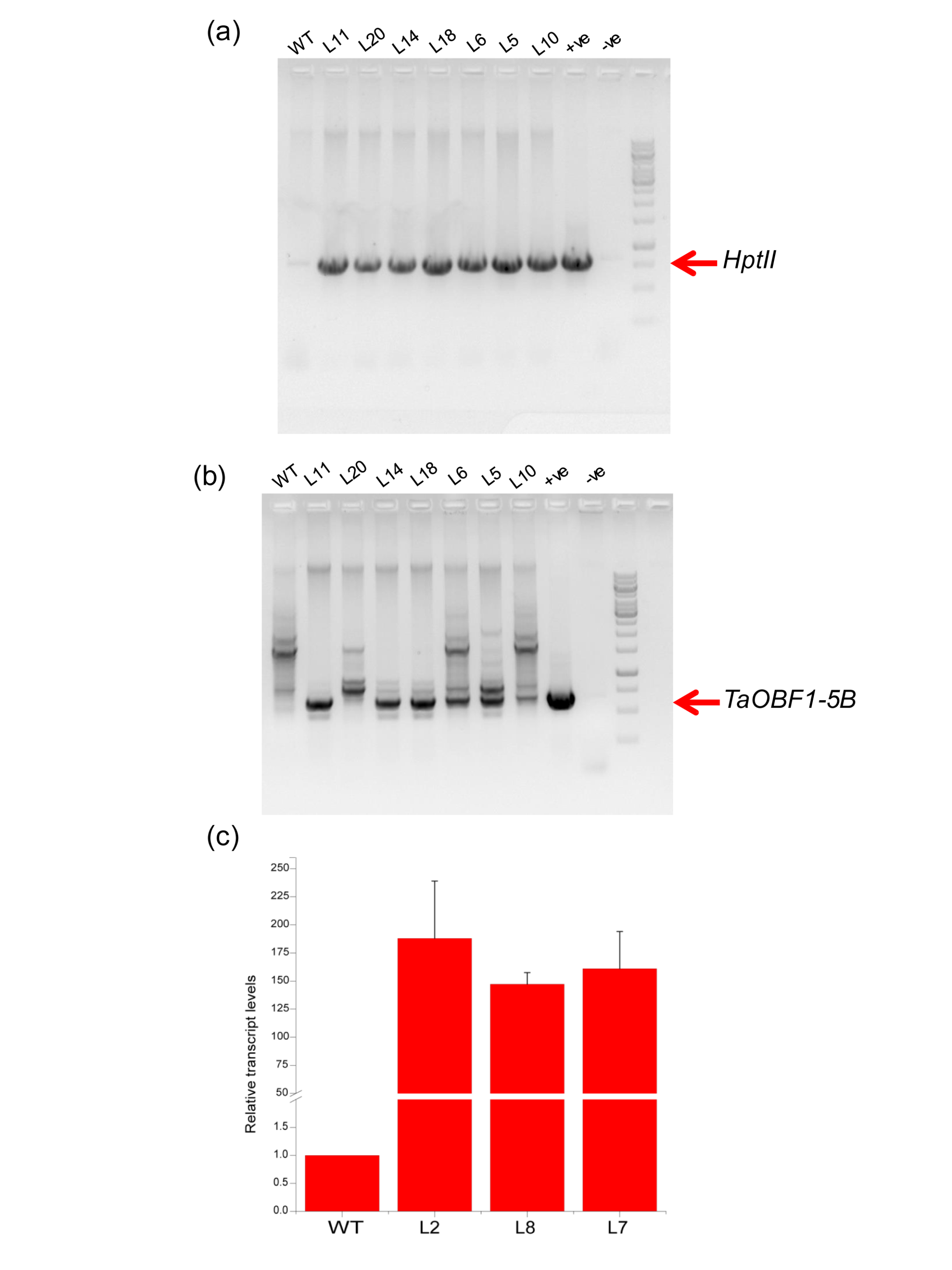

Supplement: Supplementary Figure 8 — Confirmation of TaOBF1-5B overexpression rice transgenic lines by (A) Hygromycin resistance gene specific PCR (B) Gene and vector-specific PCR (C) RT-PCR. [file Image_8.TIF]
