# Supplementary material for: Wheat ocs-Element Binding Factor 1 Enhances Thermotolerance by Modulating the Heat Stress Response Pathway
Source: Front Plant Sci. 2022 May 31;13:914363. doi: 10.3389/fpls.2022.914363 (PMC9194769; doi:10.3389/fpls.2022.914363)
Supplement: Supplementary Table 1 — List of primers used in this study. [file Table_1.pdf]

Supplementary Table 1. List of Primers used in this study

S.No	Primer Name	Primer Sequence
1	*TaOBF1-5B cDNA F*	GGTAGCAGCATCATCATCATCAGCATT
2	*TaOBF1-5B CDS F*	ATGTCGTCGTCGTCGCTGTCGCCGGGA
3	*TaOBF1-5B CDS R*	TCAGTACTGGAGCATGTGCGTGGCGGT
4	*TaOBF1-5D cDNA F*	GCTAGGCTAGGCTAGATCAGCGGGAG
5	*OsHSP100 RT F*	TGCTGGAGCAAAGGAATGG
6	*OsHSP100 RT R*	TGCCGCTGGATGAACTTCTC
7	*AtHsfA2 RT F*	ATGGAAGAACTGAAAGTGGAAATGG
8	*AtHsfA2 RT R*	TTAAGGTTCCGAACCAAGAAAACCC
9	*AtHSP100 RT F*	GAGGAGTTGCTTTGGCAGTC
10	*AtHSP100 RT R*	CAGCGCCTGCATCTATGTAA
11	*AtHSP70 RT F*	GGAAAGTTCGAGCTCAGTGG
12	*AtHSP70 RT R*	ACCTTCCCTTGTCGTTTGTG
13	*AtAPX2 RT F*	CTGGAGGACCAGAGATTCCA
14	*AtAPX2 RT R*	GTGTGTCCACCAGACAATGC
15	*AtCAT RT F*	GATCTCGGTATCCCACAGGA
16	*AtCAT RT R*	AGAGATCTTTGGTGGCATGG
17	*AtHsfA3 RT F*	TCATCTCTTGGGGACTGACC
18	*AtHsfA3 RT R*	GCCTCTAAGGAAAGCCTCGT
19	*AtHsfA6 RT F*	TGGGAACCAGAGACTTTTGC
20	*AtHsfA6 RT R*	TGCCTCTCTCCCTTCAGAAA
21	*AtActin RT F*	GATACACGCTTCCTCATGCTATCC
22	*AtActin RT R*	AGAGCCACCGATCCAGACACTG
23	*OsUbiquitin RT F*	ACCACTTCGACCGCCACTACT
24	*OsUbiquitin RT R*	ACGCCTAAGCCTGCTGGTT
